# Rapid DNA cleavage by the LINE-1 endonuclease proximal to DNA ends and at mismatches

**DOI:** 10.1016/j.jbc.2025.110994

**Published:** 2025-11-29

**Authors:** Bryant D. Miller, Benedict A. Smail, Trevor Van Eeuwen, Hanna Kodama, Kazuma Kondo, Hua Jiang, Allison O’Brien, Nan Dai, Robert J. Trachman, Shengxi Guan, Jennifer A. Karlow, Wen-Chih Cheng, John M. Sedivy, Gerwald Jogl, Michael P. Rout, John LaCava, Kathleen H. Burns, Martin S. Taylor

**Affiliations:** 1 Department of Pathology, Dana Farber Cancer Institute and Harvard Medical School, Boston, Massachusetts, USA; 2 Laboratory of Cellular and Structural Biology, The Rockefeller University, New York, New York, USA; 3 Department of Molecular Biology, Cell Biology, and Biochemistry, Brown University, Providence, Rhode Island, USA; 4 Department of Pathology and Laboratory Medicine, Brown University, Providence, Rhode Island, USA; 5 Legorreta Cancer Center, Brown University, Providence, Rhode Island, USA; 6 Brown Center on the Biology of Aging, Brown University, Providence, Rhode Island, USA; 7 New England Biolabs, Inc., Ipswich, Massachusetts, USA; 8 European Research Institute for the Biology of Ageing, University Medical Center Groningen, Groningen, The Netherlands; 9 Department of Pathology, Massachusetts General Hospital, Boston, Massachusetts, USA

**Keywords:** endonuclease, DNA conformation, DNA damage, transposable element (TE), LINE-1, retrotransposon

## Abstract

Long interspersed element 1 (LINE-1, L1) is a eukaryotic retrotransposon that propagates through an RNA intermediate. Its mutagenic insertion mechanism, target-primed reverse transcription (TPRT), requires coordinated activities of the encoded ORF2 protein (ORF2p) endonuclease (EN) and reverse transcriptase (RT) domains. EN initiates TPRT by nicking target genomic DNA, creating a 3′-OH that primes ORF2p RT for complementary DNA synthesis using the bound L1 RNA template. L1 insertions occur preferentially at 5′-TTTTT↓AA consensus motifs; this bias could reflect site-specific EN cleavage or sequence requirements in the subsequent RT priming step, in which the cut genomic DNA flap must base pair with the poly(A) RNA template. We find that, *in vitro*, EN is promiscuous, cutting linear DNA oligonucleotides and plasmids at many non-consensus sites. We discovered a cleavage activity on a mismatched substrate that was nicked ∼40-fold faster than duplex DNA containing the consensus site and identify three features promoting rapid cutting. First, EN cleaves two nucleotides downstream of mismatches, favoring A-G mismatches or T•G/U•G wobble pairs. Second, both mismatch and consensus sequences are cleaved >2-fold faster when proximal to a DNA end. Third, end-proximal EN cutting depends on end composition: 5′ overhangs cut fastest, followed by 3′ overhangs, followed by blunt ends. Together, these results indicate that EN cleavage is based primarily on DNA structure rather than sequence, that many L1 insertion attempts likely fail after cleavage at the priming step , and that mismatches and possibly other DNA conformational alterations promote EN cleavage, broadening our understanding of the genomic impact of L1.

The long interspersed element 1 (LINE-1, L1) retrotransposon has written more than one-third of the human genome through its ‘copy and paste’ mechanism (1, 2). This encompasses ∼ half a million L1 sequences mobilized in *cis* and making up ∼17% of human DNA, ∼one million *Alu* short interspersed elements (SINEs) mobilized in *trans* and making up ∼10% of human DNA, as well as other retroelements and processed pseudogenes (1, 2). It has been estimated that each person inherits ∼150 full-length retrotransposition-competent L1 elements, which include polymorphic insertion variants segregating in human populations (2, 3); a handful of these are “hot” or highly active for retrotransposition (3, 4, 5). L1 elements are transcriptionally repressed by promoter methylation and through chromatin modifications, but they become derepressed and can cause *de novo* insertions in gametogenesis, early development, and in many cancers, where numerous somatically-acquired L1 insertions can be found in tumor genomes (6, 7, 8, 9, 10, 11, 12, 13, 14, 15).

A full-length L1 RNA transcript is ∼6000 nt long and contains two ORFs that encode two proteins required for retrotransposition: the 40 kDa RNA-binding ORF1 chaperone protein (ORF1p) and the 150 kDa ORF2 protein (ORF2p). ORF2p consists of five annotated domains: the N-terminal apurinic/apyrimidinic (APE)-like endonuclease (EN) domain, which is a metal-dependent endonucleolytic phosphohydrolase (16, 17); a “tower” (18, 19) that moves together with EN to coordinate retrotransposition (18, 19, 20); the reverse transcriptase (RT) domain (18, 21); a “wrist” that binds the RNA template (18, 19); and a C-terminal domain that forms a cysteine-rich zinc knuckle with nucleic acid binding activity (19, 22, 23). ORF1, ORF2, and all their domains are required for retrotransposition (18).

L1 retrotransposes through a process termed target-primed reverse transcription (TPRT) (19, 24, 25, 26). In this model, reverse transcription of L1 RNA to complementary DNA (cDNA) is primed from a nicked target site in the genomic DNA that presents a free 3′-hydroxyl (-OH). A:T Watson-Crick base pairing between the L1 RNA poly(A) tail and the single-stranded genomic DNA flap formed from the newly formed nick primes the RT reaction that extends the 3′-OH into a cDNA flap (Fig. 1*A*). These reactions have been reconstituted *in vitro* with purified ORF2p (19, 25). Second strand synthesis and repair occur by unknown mechanisms that may be catalyzed by ORF2p, the host, or both, depending on the circumstances (27, 28, 29). An off-set or staggered nick then used to prime second strand synthesis can explain the target site duplications (TSDs) that flank new L1 insertions, but this has not been directly demonstrated with human L1 (18, 19, 24, 25, 26, 30, 31).

**Figure 1:**
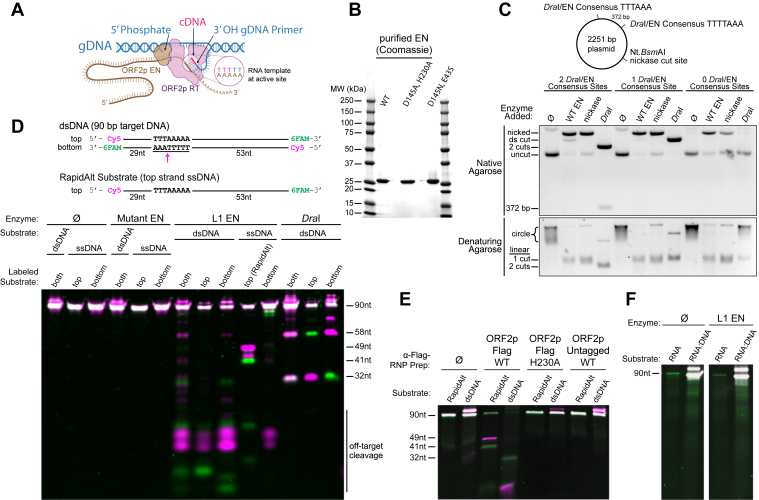
**Discovery of a robust noncanonical cutting activity**. All reactions performed in 50 mM Tris pH 7.5, 50 mM KCl, and 5 mM MgCl2. *A*, a graphic showing site of EN nicking and the A:T base-pairing thought to be important for priming synthesis of cDNA by reverse transcriptase (RT). *B*, WT; [D145A, H230A]; and [D145N, E43S] mutants were purified using Ni-NTA followed by Heparin affinity column followed by size exclusion for ultrapure product. [D145N, E43S] mutant was used for all downstream experiments. *C*, purified L1 EN activity on plasmid DNA produces a nicked (*open circle*) product. Plasmids with two consensus EN/*Dra*I cut sites (one 5′-TTTTAAA and one 5′TTTAAA), one canonical EN/*Dra*I cut site (5′-TTTTAAA), and zero canonical EN/*Dra*I cut sites showed similar rates of EN cutting activity on both native and denaturing agarose gels, revealing a lack of EN cutting specificity for the consensus site. The *Dra*I control showed the expected cutting patterns for each condition. For the denaturing gel, the sharp bands in each lane likely represent substrate that did not fully denature. Conditions: 110 nM EN and 200 ng plasmid for 1 hour. *D*, recombinant L1 EN activity on annealed double-stranded fluorescent substrates (dsDNA) or the individual oligonucleotide strands (ssDNA). 5′Cy5 and 3′6-FAM labels were present on “*top*,” “*bottom*,” or both strands of the annealed duplexes or single DNA strands as indicated. On annealed dsDNA, there is some cutting at the presumed consensus site (58 + 32 nt), as marked by the *Dra*I restriction cut (TTTAAA), and greater amounts off-target activity, with many other products formed. When the individual strands are assayed (not annealed), L1 EN produces a strong and specific cut on ssDNA+ (*top* strand, hereafter 'RapidAlt') but not ssDNA- (*bottom* strand). No activity is seen with catalytic inactive mutant EN. Conditions: 75 nM EN and 50 nM substrate for 30 min. *E*, purified L1 RNP particles display activity similar to recombinant L1 EN on fluorescent substrates; no activity is seen with catalytic null (H230A) EN or a prep where ORF2p is untagged. Conditions: 200 mg rehydrated RNP pellets, 1 mg saturated beads with antibody, 100 pmol DNA substrate in a 50 μl reaction incubated for 1 hour. *F*, L1 EN does not show activity on either ssRNA or an RNA-DNA hybrid. Conditions: 100 nM EN and 50 nM DNA substrate incubated for 1 hour. cDNA, complementary DNA; EN, endonuclease; FAM, 6-carboxyfluorescein; L1, long interspersed element 1; Ni-NTA, Nickel-Nitrilotriacetic acid; ORF2p, ORF2 protein; RNP, ribonucleoprotein.

L1 EN is essential for efficient retrotransposition, and cell culture-based assays with mutant ORF2p constructs have shown that loss of L1 EN function reduces the retrotransposition frequency to less than 1% of wild type (32, 33, 34). Residual activity (EN-independent retrotransposition) is due to L1 insertions at preexisting DNA breaks, compromised telomeres (33, 34, 35, 36), or at the 3′OH of an Okazaki fragment, similar to some ancestral mobile group II introns (18, 35, 37, 38). When EN is intact, the consensus site for insertions is a 5′-TTAAAAA (top strand), reflecting a 5′-TTTTT↓AA on the reverse complement strand nicked by EN, though variability is tolerated (35, 39). The consensus likely reflects a combination of both EN targeting preference (25, 32, 39) and the requirements for base-pairing to prime RT. Indeed, EN-independent L1 insertions still exhibit this insertion preference, albeit more weakly (35). Prior work has shown that L1 EN nicks supercoiled plasmids with this consensus site *in vitro* and determined that L1 EN shows no preference for apurinic/apyrimidinic sites (32) despite its similarity to APE1 EN (40).

L1 EN crystal structures (40, 41) and biochemical studies (39, 41) suggest that the enzyme recognizes the structure of a B-form DNA helix at the “T-A step” (40, 41, 42, 43) between opposing A tracts. At these sites, minimal stacking interactions promote access to the minor groove; this in turn permits EN-mediated minor groove compression (upstream of the cleavage site) and widening (downstream); scissile bond rotation at the cleavage site, and ultimately phosphodiester hydrolysis (41). A recurrent theme in these reports is that L1 EN recognizes features of the helical structure predominantly through interactions with the DNA sugar-phosphate backbone, not the bases themselves. Effects of helical distortions beyond the T-A step of the L1 consensus site sequence have not been explored.

Here, we sought to identify features of DNA cleavage by L1 EN. We purified the L1 EN domain to homogeneity and examined cleavage of plasmid DNA, fully base-paired DNA duplexes, and DNA fragments predicted to form mixed combinations of duplexes, mismatch bubbles, and hairpins. We identify a novel nicking activity on DNA duplexes containing a mismatch which favors W-G (W = A or T) mismatches two base pairs upstream of the cleavage site, with the W nucleotide on the strand undergoing cleavage. L1 ribonucleoprotein complexes (RNPs) from human cells containing full length ORF2p share this activity. We also found that purified EN preferentially cuts near a DNA end and downstream of a 5′ overhang. Our data suggest a generalized structure for substrates of noncanonical L1 EN cutting and that the activity depends on the generated structure rather than sequence.

## Results

### A noncanonical activity of L1 EN

We purified monodisperse L1 EN to homogeneity (residues 1–238 AA of ORF2p), along with double mutant [D145A, H230A] and [D145N, E43S] catalytically dead enzymes to be used as controls (Fig. 1*B*, Fig. S1). We tested L1 EN on a series of 2.2-kb supercoiled plasmids containing two, one, or zero consensus cut sites (5′-TTTTAAA; note that in the plasmid with two sites, one site is TTTAAA). TTTAAA is cut by the restriction enzyme *Dra*I, which was used as a control (5′-TTT↓AAA, producing a blunt-ended double strand break). We found that L1 EN nicks supercoiled plasmid DNA as originally reported (44), resulting in mobility identical to cleavage by nickase Nt.BsmAI at a separate site (GTCTCN↓, Fig. 1*C*). However, the same nicking by EN is observed in all three plasmids and does not depend on the presence of the consensus motif (Fig. 1*C*). *Dra*I cleavage shows the expected product bands for each plasmid, and denaturing urea agarose shows results consistent with the native agarose (Fig. 1*C*). These data demonstrate that supercoiled plasmid nicking by L1 EN does not require a consensus site.

We next tested cutting on a random 90 bp dsDNA oligomer containing a single consensus L1 target site (5′-TTTTTAAA) on the bottom strand. This substrate was generated by annealing two synthetic oligonucleotides that were both fluorescently labeled with a 5′-Cy5 and a 3′-6-carboxyfluorescein (FAM), resulting in a quadruply end-labeled dsDNA molecule. We used both [D145A, H230A] and [D145N, E43S] catalytically dead mutant EN as negative controls (Fig. S1), neither of which showed activity; we chose the more stable [D145N, E43S] mutant as the negative control for all subsequent reactions. As expected, L1 EN nicks this dsDNA substrate to separate a 32 nt 3′ 6-FAM labeled fragment and a 58 nt 5′ Cy5-labeled fragment on a denaturing gel, like fragments produced from the same product digested with *Dra*I restriction enzyme (Fig. 1*D*, further confirmed by dsDNA labeled on only ‘top’ or ‘bottom’ strand, generated by annealing one labeled and one unlabeled strand). However, these canonical fragments were a minor product. Most products were shorter fragments of off-target dsDNA cleavage.

We performed control reactions in parallel with these experiments in which we exposed the individual single stranded 90-mers to L1 EN (ssDNA, the top or bottom strand of the dsDNA substrate) (Fig. 1*D*). Unexpectedly, we found that L1 EN cut the “top strand” of the dsDNA consensus target (hereafter the “RapidAlt” substrate) with what appeared to be both higher rate and specificity, producing two distinct similarly sized fluorescent bands; the “bottom strand” was not cut (“ssDNA bottom”, containing the consensus EN nick site). L1 EN cutting of both substrates was somewhat inhibited at increasing KCl concentrations, with some inhibition at 150 mM in most experiments and near-complete inhibition at 500 mM, likely due to effects of the salt on substrate DNA binding (Fig. S2). These findings together indicated a new cryptic cleavage activity which we then sought to further characterize.

Given the absence of activity from catalytically dead mutants, purified identically from *Escherichia coli*, together with the high purity of this enzyme (Fig. 1*B*), we did not expect that this novel activity was attributable to a copurifying contaminant. However, to ensure that the L1 EN cleavage activity was not an artifact of separating the N-terminal EN domain from ORF2p or protein misfolding, we purified L1 RNPs from human cell lysates expressing full-length Flag-tagged ORF2p (20, 45, 46, 47) on anti-Flag-conjugated Dynabeads, adding the fluorescent substrates directly to the immobilized RNPs. As in the reactions with bacterially expressed recombinant L1 EN domain, L1 RNPs expressed in human cells and containing full-length ORF2p specifically cut RapidAlt, producing the same products seen previously on denaturing gel. However, cleavage of the dsDNA substrate resulted in loss of most of the detectable substrate, yielding a substoichiometric amount of the expected 32 nt 3′ 6-FAM labeled fragment, no expected 58 nt 5′ Cy5-labeled fragment, and a smaller Cy5-labeled band. These results are consistent with the results of purified EN domain, with some specific cutting and mostly nonspecific cleavage on dsDNA (Fig. 1*E*). No activity was seen when using L1 RNPs from cells expressing an ORF2p H230A EN catalytic mutant (32) nor from cells expressing native (untagged) ORF2p (a mock IP). These findings indicate a noncanonical L1 EN cleavage activity evident in both purified L1 EN domain and immunoprecipitated L1 RNPs with full-length ORF2p which is dependent on EN catalytic activity.

To evaluate whether the L1 EN cleavage activity was specific for DNA, we tested an RNA substrate with the same “top strand” sequence (replacing thymine for uracil) as well as an RNA-DNA hybrid (an RNA “top strand” sequence annealed with a ssDNA “bottom strand”; Fig. 1*F*). Neither the RNA alone, nor the RNA-DNA hybrid was cut.

### Sequencing L1 EN cleavage products

**Figure 2:**
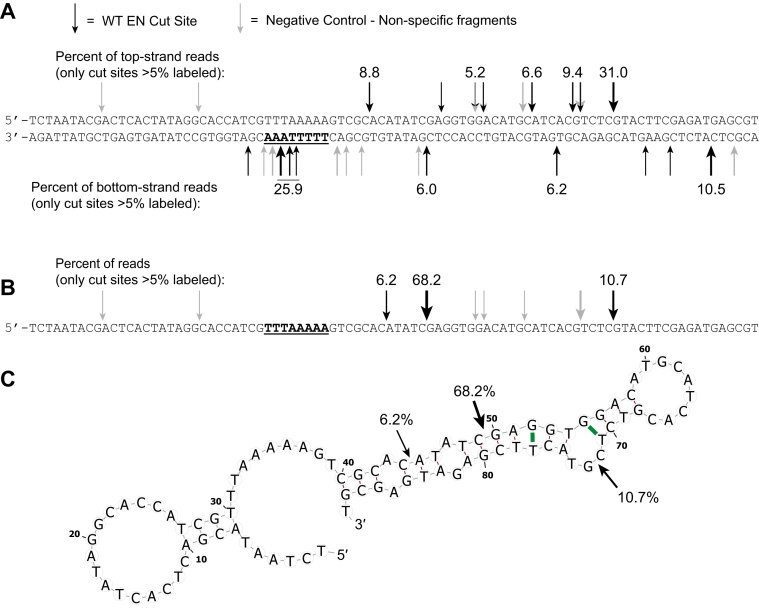
**Sequencing of L1 EN cut sites on dsDNA and self-annealed ssDNA substrates**. Reaction conditions: 100 nM substrate cut with 200 nM L1 EN (or catalytically dead enzyme) for 15 min in 10 mM Tris pH 7.5, 5 mM MgCl2, and 5 mM KCl. *A*, L1 makes a promiscuous cut at the consensus site, with ∼26% of all bottom-strand reads occurring at or near the TTTTTˆAAA site. On the top strand, 31% of cuts occurs at the same CpG site seen as the second-most common site in the RapidAlt substrate (ssDNA+). There are a variety of other cuts observed in dsDNA, with an enrichment for CpG sites, or more generally YpR sites. Negative control fragments (*gray arrows*) were generated using a catalytically dead EN enzyme [D145N, E43S] followed by the same sequencing protocol. The negative control fragments were identical for both dsDNA top strand and RapidAlt substrates (Table S2) *B*, L1 makes three specific cuts in RapidAlt (ssDNA+); the majority (∼70%) occur at an internal CpG site, ∼11% occur at a more distant 3′ CpG site and ∼6% occur at a CpA site upstream of the primary cut. *C*, predicted secondary structure of RapidAlt substrate in B with cut sites labeled. EN, endonuclease; L1, long interspersed element 1.

To determine the precise L1 EN cut sites on the dsDNA (unlabeled dsDNA “target DNA” Fig. 1*D*) and RapidAlt substrates, we performed next-generation sequencing of reaction products. For double-stranded DNA, sequencing showed that 25.9% of cuts occur at or adjacent to the T-A step (40, 43) in the 5′-TTTTTAAA site on the “bottom strand” (Fig. 2*A*), consistent with the sizes of labeled fragments seen by gel electrophoresis (Fig. 1*D*). Meanwhile, significant off-target cleavage was evident on both strands with an enrichment for CG dinucleotides [5′-C↓G or, more broadly, 5′-C↓R (C, followed by G or A) ](Fig. 2*A*, Table S1) (32, 39). In contrast, cleavage of the RapidAlt substrate was remarkably site-specific; 68% of reads indicated cleavage at a CG dinucleotide 49 nt from the 5′ end (Fig. 2*B*). A second CpG site 23 bp 3′ on the strand represented approximately 11% of cleavage sites; a third cut site, a CA dinucleotide, was reflected in 6% of the reads. No other significant cut sites (>2.1% of reads) were identified. We note one limitation of the sequencing protocol: due to a primer amplification step, the 5′ end of each strand is obscured, and so off-target cleavage close to the DNA ends is underrepresented.

Since L1 EN is not known to cut single-stranded DNA, nor would such an activity make sense based on the published crystal structures of EN bound to dsDNA (41), we used UNAFold (48) to identify possible secondary structures of the ssDNA substrate and mapped the three L1 EN cleavage sites into the predicted structure (Fig. 2*C*). Based on these predictions, we surmised that L1 EN may cut the CpG phosphate bond within a short DNA duplex in which the helical structure is distorted by a nearby mismatch.

### A mismatched duplex promotes noncanonical L1 EN cleavage

**Figure 3:**
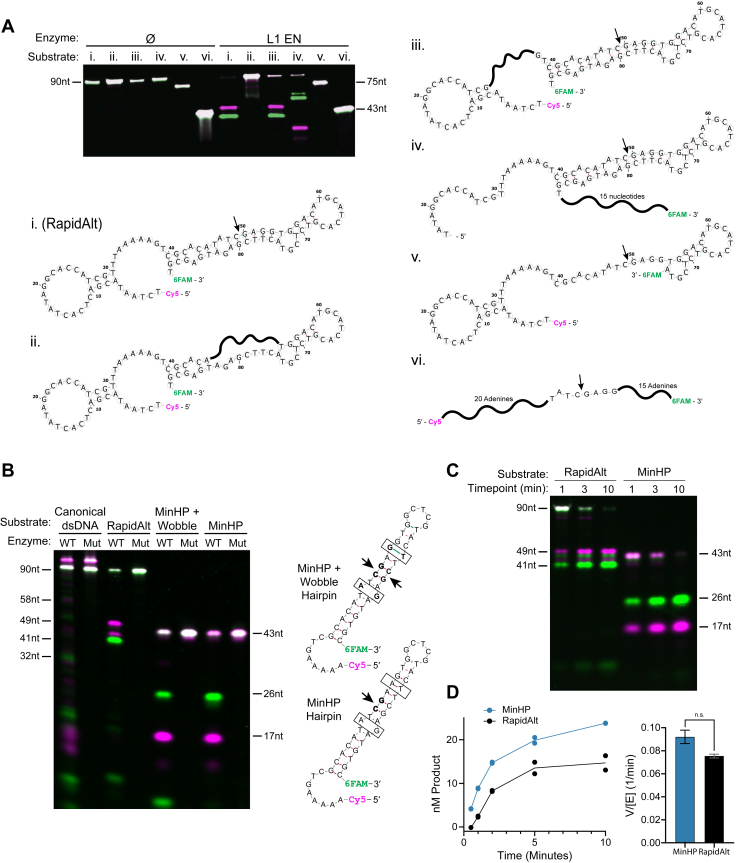
**Secondary structure enables rapid substrate cleavage by L1 EN**. *A*, DNA cleavage by L1 EN on fluorescent ssDNA substrates. i. Predited structure of RapidAlt. Scrambling of the TATCGAGG site, which is predicted to disrupt the hairpin-bulge structure (ii), removal of 15 nucleotides of the 3′ end (v), or insertion of the TATCGAGG motif into a poly-A DNA construct (vi) eliminates cutting, but scrambling the TTTAAAAA consensus site (iii) or shifting 15 5′ nucleotides to the 3′ end (iv) does not. Modifications to substrates, represented by *squiggly* lines, are idealized representations and not secondary structure predictions. Conditions: 50 mM Tris pH 7.5, 50 mM KCl, 5 mM MgCl2, 100 nM enzyme, and 50 nM substrate with 1 h incubation time. *B*, comparison of canonical dsDNA and RapidAlt substrate to two smaller hairpin substrates: one with a G•T wobble and A-G mismatch (“MinHP + Wobble”) and the other with only an A-G mismatch (hereafter called “MinHP”). Secondary structural predictions of each fluorescently labeled hairpin. *C*, comparison of RapidAlt to MinHP at multiple time points reveals that L1 EN cuts MinHP with similar rate and increased specificity compared with RapidAlt substrate. Reaction conditions for B and C: 10 mM Tris pH 7.5, 50 mM KCl, 5 mM MgCl2, and 100 nM substrate with an incubation time of 20 min. *D*, curves showing nM product of each substrate at five time points. Quantified from time course gels shown in Figure S5 and calculated using a fit to the curve generated by standards. V/[E] values for each enzyme determined using the first three time points (0.5, 1, and 2 min) for initial rates and calculated using simple linear regression (r^2^ = 0.984 for MinHP, 0.998 for RapidAlt, error bars represent standard error). *p* value = 0.1945 using a two-tailed Welch’s *t* test. EN, endonuclease; L1, long interspersed element 1.

To evaluate sequence and structural determinants of the L1 EN activity, we next made a series of modifications to the RapidAlt substrate (substrate i., Fig. 3*A*). Randomizing the eight nucleotides at the cut site from 5′-TATC↓GAGG to 5′-GCGCCTAG (Fig. 3*A*, substrate ii.) abolishes cutting altogether, while mutation of the T-A step site from 5′-TTTAAAAA to 5′-CACAGGTT (Fig. 3*A*, substrate iii.), does not affect L1 EN cleavage. These results demonstrate that L1 EN cleavage on RapidAlt does not depend on the existence of a canonical recognition site but does depend on sequence near the noncanonical cut site. Shifting the location of the cut site by moving 15 bases from the 5′ end to the 3′ end of the strand (Fig. 3*A*, substrate iv.) causes the cut product sizes to be shifted without loss of activity, while removal of 15 bases from the 3′ end, which would remove the predicted hairpin-bulge, prevents L1 EN activity (Fig. 3*A*, substrate v.). This indicates that nucleotides at the 3′ end of the strand are important for cleavage despite their distance in sequence space from the actual cut site. Consistent with the hypothesis that structural context around the cut site is critical for the activity, no cutting was seen when the 5′-TATCGAGG sequence is flanked with A homopolymers (Fig. 3*A*, substrate vi.).

Since L1 EN is evolutionarily related to the APE ENs, we tested APE1 EN activity against both the canonical and RapidAlt L1 EN substrates (Fig. S3). APE1 activity is limited to a synthetic abasic site, consistent with its known function (49). L1 EN does not cut the abasic site (Fig. S3) as previously reported (32, 39).

We next sought to generate a minimal substrate that could recapitulate the rapid cutting seen in RapidAlt and thus constructed a series of deletions of the 90-mer to identify a shorter oligonucleotide that was still effectively cut. We progressively reduced the 90-mer to 43 nucleotides, preserving the base pairing and nucleotides at the cut site but eliminating much of the 5′ end and converting the 3′ end to a “clean” hairpin retaining the A-G mismatch, with or without the downstream at G•T wobble pair from RapidAlt. Fluorescently labeled versions of these minimal substrates showed specific cleavage after both the A-G mismatch and G•T wobble at rates faster than the canonical TTTTT↓AA substrate and similar to RapidAlt. The substrate with both the wobble and mismatch was cleaved twice while the mismatch-only substrate was only cleaved once (Fig. 3*B*). We name the minimal hairpin substrate with a single A-G mismatch “MinHP” and observed that EN cuts MinHP at a similar rate but with increased specificity compared to the RapidAlt substrate (Fig. 3*C*, Fig. S4). To verify this result, we performed a time-course experiment and calculated the appearance of cleaved product band using a standard curve. We then used the first three time points to estimate EN cleavage rate for each substrate. MinHP was cut slightly faster than RapidAlt, although the difference was not statistically significant: V/[E] 0.092 *versus* 0.075 per minute, respectively (Fig. 3*D*, Fig. S5). Collectively, these results demonstrate that L1 EN cuts MinHP 2 bp downstream of the A-G mismatch with greater specificity than RapidAlt and at a rate comparable to RapidAlt, making it a good minimal substrate.

### L1 EN mismatch cutting depends on an A-G or T•G mismatch

**Figure 4:**
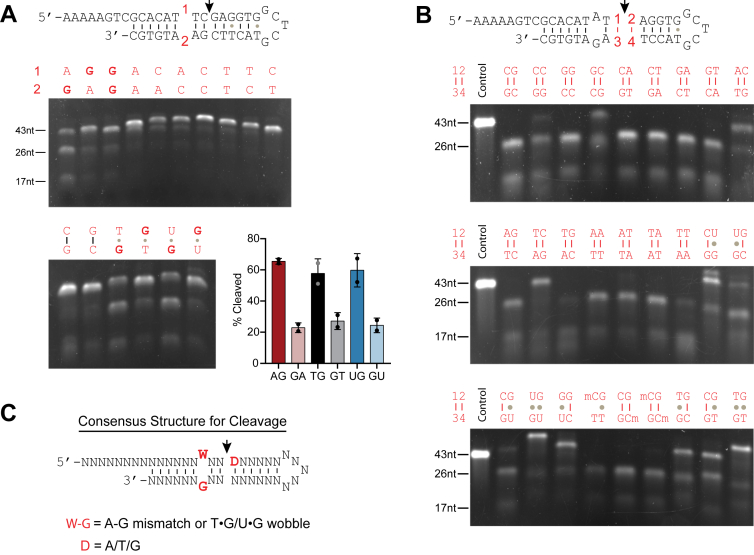
**Pinpointing noncanonical L1 EN cleavage specificity**. *A*, L1 EN activity on structures with all possible canonical mismatched nucleotides. The first letter corresponds to (1) and the second to (2) in the structure in *panel A*. Quantification reveals a roughly 60% cleavage rate for AG, TG, and UG indicating that a mismatched G in position (2) facilitates efficient cleavage (*error bars* represent standard deviation). The reverse pairs are cut ∼3-fold less efficiently and very little cutting occurs when the mismatch is replaced with a full base pair (not quantified). The majority of other mismatches do not permit cutting. Percent cleavage was calculated by dividing the intensity of both substrate bands by the total lane intensity. *B*, L1 EN activity with the A/G mismatch held constant and all possible canonical mutations of the four nucleotides at the cut site plus structures with methylated or deaminated cytosines. Many of these sequences are cut, indicating fewer sequence requirements for the cleaved sequence in comparison to the mismatch that forms the bulge. Methylation of one or both Cs proximal to the cut site still permits cutting, but deamination of one or both of these Cs blocks it. Substrates with a *C* in position (3) are cut poorly. Conditions for A and B: 50 mM Tris pH 7.5, 5 mM MgCl2, 50 nM enzyme, and 50 nM substrate with an incubation time of 1 h. *C*, consensus structure cut by L1 EN. EN, endonuclease; L1, long interspersed element 1.

Having established that L1 EN makes a single-stranded cut at a 5′-C↓G dinucleotide two base pairs downstream of an A-G mismatch and G•T wobble, we next investigated features of the mismatched bases that affect this activity. First, we mutated the mismatched pair in a hairpin substrate with an A-G mismatch to 16 different combinations, including all 12 non-Watson–Crick pairs, two matched C-G pairs, and 2 G•U pairs (deoxyuridine substituted for cytosine). We chose to include both dG•dU and dG•dT wobble pairs that occur commonly in DNA both sporadically (2) and in carcinogenesis (50) due to cytosine and CpG 5-methylcytosine deamination, respectively (51, 52). Of these substrates, the A-G mismatch and T•G and U•G wobbles are all efficiently cut. Of note, the orientation of the mismatch appears to matter. An A-G mismatch results in nearly 3-fold more cleavage than a G-A mismatch, with similar differences seen in [T•G] > [G•T] and [U•G] > [G•U] wobble pairs (Fig. 4*A*, Fig. S6). Little or negligible cleavage was detected with any of the remaining mismatches, with essentially no cutting at pyrimidine-pyrimidine mismatches and a small amount of cutting with a G-G mismatch (Fig. 4*A*). These results show that L1 EN cleavage on a mismatched substrate is strongest when the mismatch is either an A-G mismatch, a T•G wobble, or a U•G wobble pair.

### L1 EN cleavage of mismatched substrates tolerates degenerate sequences at the cut site

We next investigated whether there are nucleotide requirements for cleavage at the cut site. We did this by substituting the two base pairs flanking the hydrolyzed bond on a hairpin substrate containing a single A-G mismatch (Fig. 4*B*). Selected mutations included 26 combinations—all 16 canonical Watson–Crick pairs; five pairs containing deoxyuridine substitution(s); three pairs in which the C’s at the cut site were methylated; and three pairs in which one or both C’s were replaced with T’s. Most of these oligonucleotides are effectively cut by L1 EN, suggesting that base pairs closest to the cleavage site are not restrained (Fig. 4*B*). One exception is the placement of a C immediately 3′ of the cut phosphodiester bond, which inhibits cutting, especially if the prior nucleotide is an A or a T. The replacement of a deoxycytosine proximal to the cut site with deoxyuridine and the introduction of T•G wobble pair also inhibits cutting. The presence of methylated cytosine at the CpG cut site has no impact on cleavage. (Table S2). Thus, except in a few cases, there is little sequence requirement precisely at the cut site, in contrast to the requirement at the mismatched position 2 nt upstream. Our data suggest a generalized structure for substrates of noncanonical L1 EN cutting (Fig. 4*C*), which allows a wide range of possible substrates provided the existence of the W-G mismatch.

To confirm that L1 EN cleavage depends on the presence of the mismatch but not on the sequence at the cut site or on the hairpin structure, we designed five annealed duplex structures that preserve many features of the original substrate (Fig. 4*C*) while randomizing intervening sequences. L1 EN efficiently cleaved all substrates with a mismatch (Fig. S7*A*). However, when we replaced the bottom strands of these constructs to eliminate the mismatch and create fully base paired DNA, cutting efficiency and specificity decreased substantially (Fig. S7*B*). These results confirmed that the mismatch, and not the sequence or the hairpin context, is the defining feature of this type of cleavage substrate.

In addition, these results can explain why only the “top” strand (RapidAlt), but not the “bottom” strand from the original single-stranded 90-mers was cut by L1 EN. The major predicted secondary structures from UNAFold for the “bottom” strand contain T-T and C-T mismatches, which are not cleaved in our data (Fig. S8). There is no predicted A-G mismatch; for the only T-G wobble predicted, the T is followed by xT↓C, which is one of the few sequences that strongly inhibits cutting (Fig. 4*B*, Fig. S8).

### Mass spectrometry validation of mismatch-induced cleavage

**Figure 5:**
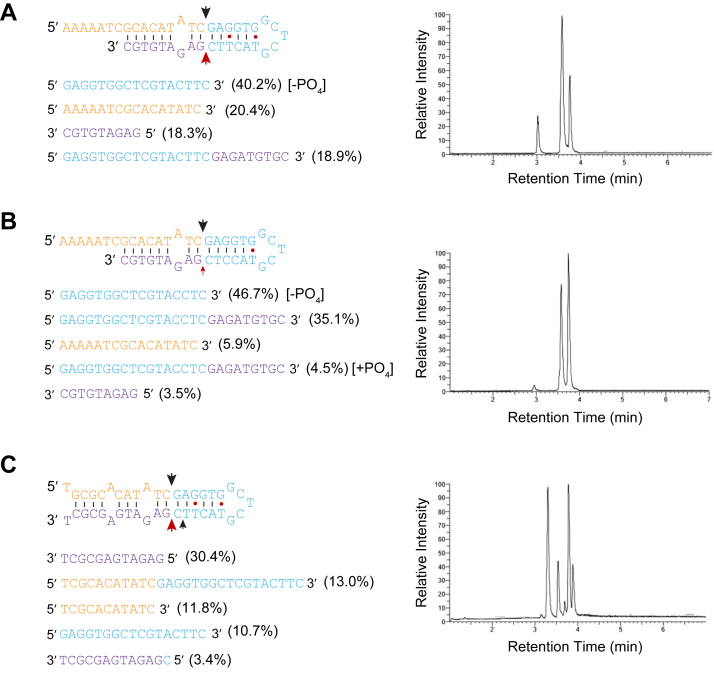
**Mass spectrometry of three L1 EN-cut DNA hairpins**. *A*, most cleavage occurs at the TATCˆGAGG site seen in sequencing (*heavy black* arrow, “*top*” strand in diagram), consistent with cleavage two bases downstream of the A-G mismatch on the “*A*” strand. Recognition of the G•T wobble 2 nt downstream of this cleavage site would result in cleavage at the same site but on the opposite strand, cutting the CTTCˆGAGA bond (*heavy red* arrow, “*bottom*” strand in the diagram). *B*, removal of the G•T wobble reduces this “*bottom* strand” cleavage (*small red* arrow) as evidenced by reduced CGTGTAGAG “*purple*” product detected. *C*, addition of an A-A mismatch does not result in cleavage around this site. Note the G•T wobble is still present in this substrate *C*. We interpret the relative lack of detected AAAAATCGCACATATC “*orange*” product in all panels to nonideal behavior in the mass spectrometer and/or nonspecific cleavage, resulting in products too small to identify. Reaction conditions: 50 mM Tris pH 7.5, 5 mM MgCl2, 50 mM KCl, 120 nM enzyme, and 80 nM substrate with an incubation time of 1 h. EN, endonuclease; L1, long interspersed element 1.

To further evaluate L1 EN cleavage of hairpin structures, we carried out liquid chromatography-mass spectrometry analysis (LC-MS) on L1 EN treated substrates. L1 EN cut efficiently at the phosphate bond two bases away from an A-G mismatch and T•G wobble, but not an A-A mismatch, confirming the gel-based results (Fig. 5). Removal of the T•G wobble reduces the cleavage 2 nt downstream of the T (compare red arrows, Fig. 5, *A* and *B*).

### EN cuts RapidAlt substrate over 40-fold faster than the EN consensus site

**Figure 6:**
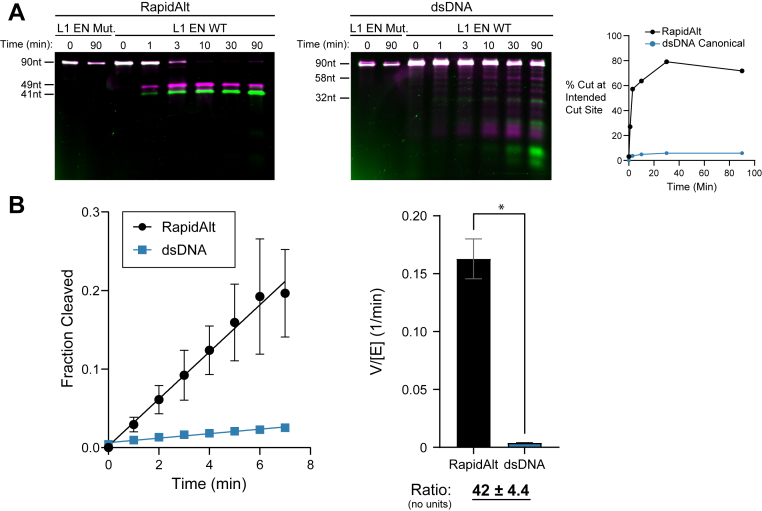
**EN cuts RapidAlt substrate over 40-fold faster than the EN consensus site**. *A*, RapidAlt substrate cutting proceeds very quickly, with complete cutting in 10 min, while the canonical sequence remains largely uncut at 90 min. Percent cleavage was calculated by dividing the intensity of the product band by the total lane intensity in the Cy5 channel. Conditions: 50 mM Tris pH 7.5, 15 mM KCl, 5 mM MgCl2, 50 nM enzyme, and 50 nM substrate. *B*, L1 EN cleaves a mismatched ssDNA substrate at a rate at least an order of magnitude faster than the consensus site in dsDNA (50 nM fluorescent substrate, 9.2 nM EN (mismatched ssDNA), 36.8 nM EN (dsDNA)). The reactions (3 total replicates) are linear with time in these conditions, and rates are calculated using simple linear regression (r^2^ = 0.80 for mismatched RapidAlt and 0.95 for dsDNA, error bars represent standard error). *p* value = 0.0115 using a two-tailed Welch’s *t* test. When corrected for the 4-fold higher enzyme concentration, the relative rate is 42.4-fold higher for mismatch cleavage. dsDNA, double-stranded DNA; EN, endonuclease; L1, long interspersed element 1; ssDNA, single-stranded DNA.

In addition to specificity, we observed apparent increased rate of L1 EN RapidAlt substrate nicking in our initial experiments as compared to dsDNA, with near complete conversion of substrate into product over the 30 min reaction (Fig. 1*D*). To further evaluate cleavage rates, we conducted time course experiments which revealed complete cutting of the RapidAlt substrate in 10 min and near-complete cutting at 3 min. In contrast, L1 EN cleavage of 90 bp duplexed DNA containing the canonical sequence remained incomplete at 90 min (Fig. 6*A*, Fig. S9). To quantify this rate difference, we performed a time course of L1 EN cleavage on the same substrates and resolved the products and substrates using capillary electrophoresis with detection of 6-FAM labels. When mismatched DNA or dsDNA (50 nM) is treated with L1 EN at 9.2 nM, there is a linear reaction rate over the first 7 min. Because the reaction rate for dsDNA is much slower, we increased the enzyme concentration 4-fold in subsequent repeat reactions. The data show that L1 EN cleavage of the RapidAlt substrate occurs at a rate approximately 40-fold faster than the consensus dsDNA substrate (Fig. 6*B*).

### A mismatch alone is insufficient for maximal cutting rate

**Figure 7:**
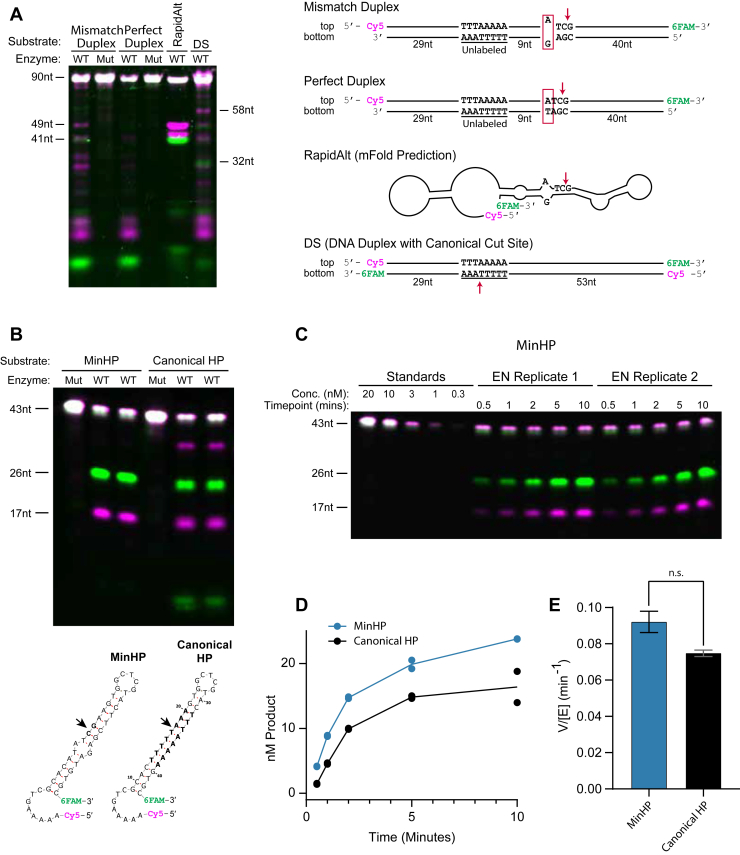
**L1 EN cuts mismatches at a similar rate to the canonical EN cut site under analogous conditions**. Reaction conditions for all experiments: 10 mM Tris pH 7.5, 5 mM KCl, 5 mM MgCl2, and 100 nM substrate. *A*, a single mismatch in the context of a full duplex (*left* lane) shows similar cutting activity to the canonical cut site in the same dsDNA context (*right* lane). In contrast, the EN cuts the RapidAlt substrate faster and more specifically. Potential cutting at canonical cuts sites in both Mismatch duplex and perfect duplex substrate is not captured because the *bottom* strand is unlabeled. Enzyme concentration: 200 nM with 25 min incubation time. *B*, summary gel showing a comparison of 2-min time points of both substrates in duplicate. More off-target cleavage is seen in canonical HP than MinHP. Controls without a mismatch showing almost no cutting are on the *right*. Enzyme concentration 75 nM with 2 min incubation time. *C*, representative fluorescent gel showing the MinHP time course assay used to calculate enzyme rates. The 6FAM (*green*) channel from this gel image, along with those in Figure S11, were used to plot the graphs in *D*–*E*. Enzyme concentration 75 nM for *C*–*E*. *D*, a graph showing nM product *versus* time for both substrates as determined by titrated standards. *E*, quantified from time course gels shown in Figure S10 and calculated using a fit to the curve generated by standards. V/[E] values for each enzyme determined using the first three time points (0.5, 1, and 2 min) in *D* for initial rates and calculated using simple linear regression (r^2^ = 0.984 for MinHP, 0.998 for canonical HP, *error bars* represent standard error). *p* value = 0.1873 using a two-tailed Welch’s *t* test. Results parallel overall cutting amount seen in B. EN, endonuclease; FAM, fluorescein; L1, long interspersed element 1.

To test the relative contributions of the mismatch and other substrate features to the specificity and rate of the RapidAlt and MinHP reactions, we designed a 90 base pair duplexed DNA substrate with a single A-G mismatch followed by a 5′-C↓G dinucleotide two base pairs downstream. The mismatch was located at the same position in the top strand as the mismatch in the RapidAlt substrate—49 nt from the 5′ end and 41 nt from the 3′ end, such that cleavage of the “top strand” would result in the same sized products as cleaved RapidAlt. We compared the specificity and rate of cleavage on this mismatched substrate to a perfect duplex, the RapidAlt substrate, and the original double-labeled dsDNA sequence (Fig. 1*D*). The mismatched duplex showed a specificity and rate similar to those of the canonical cut site—far slower and less precise than the RapidAlt substrate (Fig. 7*A*). This revealed that aspects of the RapidAlt and MinHP substrates in addition to the mismatch were important for both increased rate and specificity. To further interrogate the relative rates of EN mismatch cutting and consensus site cutting, we designed a fluorescently labeled hairpin identical to MinHP, with the EN consensus cut site replacing the mismatch (hereafter “Canonical HP”). An initial assay showed similar cutting rates for each substrate but reduced specificity cutting Canonical HP (Fig. 7*B*), with <9 nt 6-FAM-labeled products and a 35 nt 5′Cy5-labeled product consistent with off-target cutting near the 3’ end of the substrate. We then performed time course assays on both hairpin substrates (Fig. 7*C*, Fig. S10) and quantified the 6FAM fluorescent product band relative to a standard curve to measure the appearance of cleaved product over time (Fig. 7*D*). We used the near-linear first three time points (0.5,1, and 2 min) to estimate the reaction rate (Fig. 7*E*). MinHP was cut slightly faster than Canonical HP, although the difference was not statistically significant (V/[E] 0.092 and 0.075, respectively). Together, these results indicate that L1 EN cuts either a mismatch or the consensus site in an identical context, but mismatch cutting specificity is higher and the cleavage rate is slightly faster.

### L1 EN preferentially cuts near a DNA end and 5′ overhang

**Figure 8:**
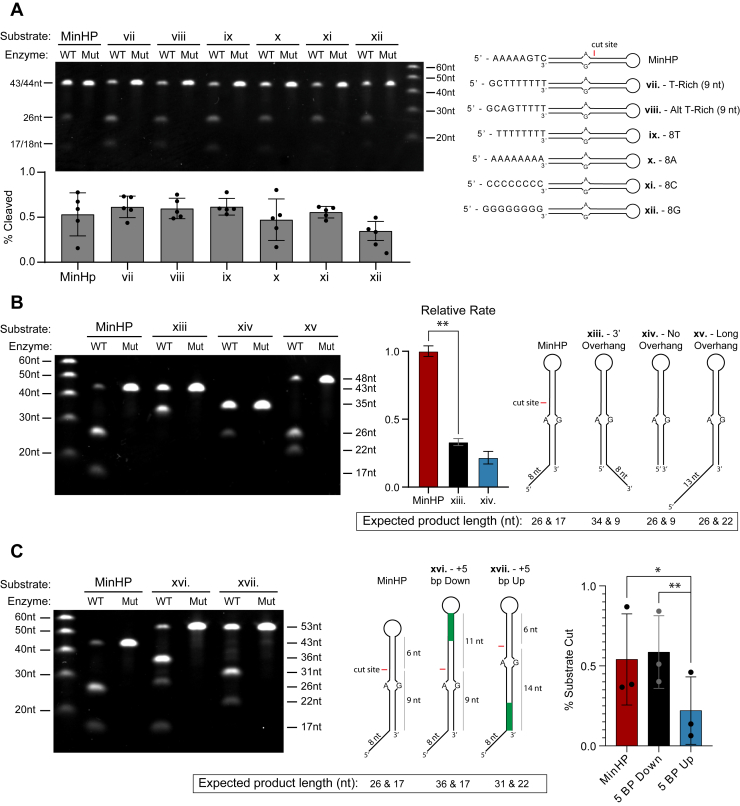
**L1 EN cutting is affected by proximity to a DNA end and overhang position (5′ *versus* 3′ *versus* no overhang) but not overhang sequence**. *A*, none of the overhang sequence modifications, including two published fast cutting sequences, poly-A, poly-T, poly-C, and poly-G sequences were cut significantly differently from MinHP (*p* values all > 0.25 using two-tailed ratio paired *t* test). *B*, summary gel showing longer overhang (13 nt) cut at similar rate to MinHP. In contrast, 3′ overhang and no overhang hairpins were cut 3-fold and 5-fold less efficiently than MinHP respectively. Relative rates were calculated by dividing the product bands by the total intensity in each lane in a 5-time point time course experiment (two replicates for each condition, Fig. S13) Rates for each enzyme determined using the first three time points (0.5, 1, and 2 min) for initial rates and calculated using simple linear regression (r^2^ = 0.994 for MinHP, 0.977 for 3′ OH, 0.846 for no OH, *error bars* represent standard error). *p* value = 0.0084 using two-tailed Welch’s *t* test. *C*, adding five bp downstream of the cut site in MinHP has no effect on cutting efficiency, while adding five bp upstream of the cut site (lengthening the distance from the end) decreases EN cutting by ∼2.5-fold (two-tailed paired *t* test). Percentage cleaved was calculated by dividing the intensity of substrate bands by the total intensity in each lane. Reaction conditions for *A*–*C*: 50 mM Tris pH 7.5, 5 mM KCl, 5 mM MgCl2, 100 nM substrate, and 100 nM enzyme with an incubation time of 10 min. EN, endonuclease; L1, long interspersed element 1.

After the discovery that a mismatch alone was insufficient to induce the rapid cleavage seen in the RapidAlt substrate, we attempted to pinpoint other secondary structural features important for rapid cutting. Recent work using full-length ORF2p showed more efficient cutting near DNA ends on substrates with a specific T-rich overhang (19). Using our isolated L1 EN enzyme, we tested a series 5′ overhang sequences in the context of the MinHP substrate. None of the overhang sequences were cut significantly differently from MinHP (A-rich), including poly-A, poly-T, poly-C, or poly-G overhang substrates and two published T-rich overhang substrates previously shown to result in accelerated cleavage (19) (Fig. 8*A*, Fig. S11). We next modified the overhang position and length on a series of hairpin substrates, including a longer overhang (13 nt), a 3′ overhang, and no overhang conditions. The longer overhang was cut at a similar rate to MinHP. However, moving the same 8 nt overhang to the 3′ end reduced cutting efficiency nearly 3-fold, and removing the overhang entirely to make a blunt substrate reduced cutting efficiency by nearly 5-fold (Fig. 8*B*, Fig. S12). We confirmed these features using short duplexed DNA oligos in place of hairpins: we used a “top” strand labeled at the 5′ end with Cy5 and annealed several unlabeled bottom strands to create substrates for L1 EN cutting. This duplex structure resembles MinHP except that the closed hairpin segment is replaced with five base pairs. Consistent with our hairpin results, filling in the 5′ end to eliminate overhanging ssDNA in the structure reduces EN cleavage efficiency (Fig. S13).

Given our previous results on various duplexed DNAs (Fig. 6*C*, Fig. S13), and recently published data from TPRT reconstitution (19), we postulated that L1 EN might preferentially cut near a DNA end. To test this, we designed two additional hairpin substrates, one with five additional base pairs upstream of the A-G mismatch in MinHP and another with 5 bp downstream of the A-G cut site. Adding base pairs downstream of the cut site had no effect on cutting efficiency. However, adding nucleotides upstream of the cut site, and thus increasing distance from the cut site to a 5′ end, reduced cutting efficiency by roughly 2.5-fold (Fig. 8*C*, Fig. S14). This observation is bolstered by the fact that most of the off-target cleavage seen in our dsDNA experiments appeared near the end of the substrate (Fig. 1, *D* and *E*; 3, *B* and *C*; 6*A*; 7, *A* and *B*). Taken together, these data demonstrate the secondary structural features necessary to achieve optimal L1 EN cutting rate and specificity. Necessary features are an A-G, T•G, or U•G mismatch or the 5′-TTTTT↓AA consensus cleavage site; proximity to a 5′ end; and a 5′ ssDNA overhang.

## Discussion

L1 EN demonstrates its canonical nicking activity at the consensus sequence of ORF2p-mediated retrotransposition events, 5′-TTTT↓AA on duplexed DNA, and has been known to nick other sites, particularly on supercoiled plasmid substrates (25, 32). In the present study, we confirm on multiple kinds of substrates that EN is only modestly specific for its consensus sequence. In addition to the consensus cleavage, CpG dinucleotides (5′- C↓G nicks) on linear matched duplexes are also prominent noncanonical cut sites, and other sequences are also cut, particularly near DNA ends. We report a potent and previously unknown L1 EN nicking activity on a DNA duplex containing a W-G mismatch (A-G, T•G, U•G) two base pairs upstream (−2 nt) of the cleavage site, where the W nucleotide is preferentially on the cut strand. There is little sequence specificity for the two bases flanking the scissile bond. Our results show that mismatched substrate cutting occurs at a rate slightly faster than that of canonical site nicking on a matched DNA duplex. Mismatched substrates are cleaved by both a recombinant EN domain fragment and by full-length ORF2p in immunoprecipitated L1 RNPs. In addition, proximity to a DNA end upstream of the cut strand in both mismatched and consensus site cutting increases the rate of nicking by an order of magnitude. This increased cutting rate occurs in proximity to blunt ends, 3′ overhangs, and hairpin bubbles, but is fastest in proximity to 5′ overhangs (∼3-fold faster than 3′ overhangs and ∼5-fold faster than blunt ends). Importantly, our data support EN cutting of dsDNA only; we identified no cuts in segments of ssDNA. Together, these data support the hypothesis that EN cleavage recognizes the structure of the target DNA rather than sequence.

The slow rate (and lack of turnover) on perfectly complementary DNA, even at the canonical cut site, could reflect slower binding, catalysis, or product release. Slow dissociation of EN from a nicked canonical cut site has been previously inferred by the deep DNA binding cleft of the protein (41), and this may allow EN to act as an “anchor”, holding ORF2p at the cut site through the multistep insertion process (18).

DNA mismatches are commonplace in cells, originating from chemical modifications (53) and polymerase errors (54, 55), especially from more error-prone DNA polymerases such as the translesion synthesis DNA polymerase eta (56, 57). Our data show U•G and T•G wobbles resulting from cytosine deamination create potent substrates for EN. Mismatch instances may be high in some cancers, especially those with higher tumor mutational burden owing to loss of mismatch repair (microsatellite instable-high cancers with loss of mismatch repair proteins MSH2, MSH6, MLH1, and PMS2) or loss of POLE (58, 59, 60). L1 EN may cause DNA damage by converting mismatches to DNA nicks and breaks, or by initiating retrotransposition at these sites. Retrotransposition appears to be an inefficient process, with many DNA damage foci seen by gamma-H2AX stain per *de novo* insertion event in L1 overexpression studies (61). EN cleavage of naturally occurring DNA lesions could explain some of this excess damage, especially if they subsequently fail to prime an RT reaction because of incompatibility of base pairing between the cut sequence and the poly(A) RNA in the ORF2p RT active site (18, 62).

In addition to cutting mismatches, our data show an increased rate of cleavage proximal to a DNA end, extending recent results from Thawani *et al*. (19), who observed that full-length ORF2p preferentially nicks and completes TPRT proximal to long 5′ overhangs, with faster cutting and more TPRT in T-rich overhang sequences (19). We confirm faster cleavage with a 5′ overhang, although in our hands the overhang sequence has little effect. L1 retrotransposition occurs mostly during S phase, and L1 preferentially inserts following EN cleavage of the lagging strand in several models (35, 36, 46, 63, 64, 65). Taken together with interactomics and genetic screening data, L1 target selection appears to occur in the context of interactions with the replication fork and replication-coupled DNA repair (13, 20, 27, 28, 46, 63). Thus, faster cleavage near DNA ends (and especially with 5′ overhangs) lends mechanistic credence to a model in which initial ORF2p EN cleavage occurs preferentially around Okazaki fragments at the replisome (19), potentially starting from mismatches introduced by polymerase alpha and delta or other distortions of the DNA helix.

The mismatch cleavage activity and our modeling together point to DNA structure, rather than sequence, as the key determinant of EN recognition and cleavage (40, 41). This novel activity may play a role in the resolution of TPRT intermediates into fully integrated genomic L1 insertions by promoting second strand cutting. Critically, completed L1 insertions are flanked by hallmark ∼12 to 18 bp TSDs, likely generated through an off-set or staggered break in the second “top” DNA strand (16, 30) potentially catalyzed by L1 EN. Although there is a strong sequence signature for the insertion site (*i*.*e*., the first strand cut and RT priming site), the second strand cut site that determines the other boundary of the TSD does not have a strong consensus sequence. The second strand cut site choice is therefore likely dictated by structural constraints. Recent studies have shown that ORF2p is extensively positively charged (19, 66) and multiple domains interact with the negatively charged sugar-phosphate backbone of the target DNA (67). We speculate that ORF2p likely distorts its target DNA into a conformation that facilitates rapid second strand cleavage by EN to enable the transition to second strand synthesis. New structural studies and perturbations that separate canonical and noncanonical L1 EN activities will be needed to investigate this.

## Experimental procedures

### Purification of L1 EN

L1 EN (amino acids 1–238 of L1 ORF2p) from human codon-optimized ORF2p (68) was prepared as previously described (32, 40, 44) with modification. L1 EN and mutants were fused to an N-terminal His-10-SUMO tag, grown in Terrific Broth containing 50 μg/ml kanamycin and 200 μl/L Antifoam 204 (Thermo Fisher Scientific) until *A*_600_ reached 1 to 3, induced with IPTG at 0.2 to 0.4 mM, grown overnight at 16 °C, pelleted, and snap frozen in liquid nitrogen. Pellets were resuspended, lysed in an emulsifier (Microfluidizer, Microfluidics), clarified, purified by Ni-NTA chromatography, tag-cleaved with purified ULP1-R3 (69), further purified by heparin chromatography, polished on size exclusion in a buffer containing 20 mM Hepes pH 8.0, 500 mM NaCl, and 0.5 mM TCEP (tris(2-carboxyethyl)phosphine), and concentrated to ∼100 to 1000 μM, depending on the construct. Aliquots of L1 EN were snap frozen and stored at −80 °C. For use in reactions, individual aliquots were diluted to 10 μM in reaction buffer (50 mM Tris pH 7.5, 50 mM KCl, and 5 mM MgCl_2_) and stored at −80 °C in single-use aliquots.

### L1 RNP reactions

L1 RNP reactions were performed from RNPs purified from cryo-milled HEK293 cell powder by anti-Flag affinity, which was prepared as described previously (70, 71). Cell powder was stored in a −80 °C freezer. For reactions, cell powder was removed from the −80 °C and placed into liquid nitrogen. For each reaction, approximately 150 mg of cell powder was weighed into a 1.5 ml tube. Cell powder was resuspended in 600 μl of RNP extraction buffer (20 mM Hepes pH 7.4, 500 mM NaCl, 1% v/v Triton-X-100, and protease inhibitors) and placed on ice for 10 min. Each suspension was vortexed until all visible clumps were gone and the solution was homogenous. Solutions were sonicated on a Misonix 3000 sonicator (Cole-Parmer) at setting five for 3 s, followed by 5 s pause, and then 3 s again, and then spun for 10 min at 20,000 rcf and 4 °C. The supernatant was extracted and mixed with 10 μl of anti-Flag rigid 2.8 micron paramagnetic beads (Dynabeads), prepared as described previously (72). The solution with beads was vortexed to make the solution homogenous. Bead-supernatant mix was incubated on a Roto-Mini rotator (Benchmark Scientific) at setting 20 for 30 min at 4 °C. After 30 min, each solution was placed on a magnet and the supernatant extracted. Beads were washed 3 times with 1 ml of extraction buffer, vortexing after each wash and putting back on the magnet. During the third wash, all solutions were transferred to fresh tubes. After the third wash, a final wash was performed with 200 μl 1X RNP TPRT Buffer (20 mM Hepes pH 7.4, 10 mM KCl, and 2.6667 mM MgCl_2_). Bead solutions were vortexed, placed on the magnet, and all supernatant and bubbles removed. Each tube of beads received 20 μl of reaction solution containing 0.5 pmol fluorescent DNA substrate and 1X RNP TPRT Buffer. Tubes were vortexed well to mix and incubated in a ThermoMixer (Eppendorf) for 1 h at 37 °C while shaking at 1000 rpm. After reaction was complete, 20 μl of 98% formamide, 10 mM EDTA was added directly to the beads and vortexed well. The tubes were then placed on a magnet and the entire supernatant was extracted (40 μl) and 20 μl of each solution was loaded onto a 10% or 15% PAGE, 7M urea denaturing gel.

### Reaction conditions

Reactions with recombinant EN (1–238 of L1 ORF2p) were carried out in either 10 mM or 50 mM Tris pH 7.5 and 5 mM MgCl_2_ with 0 mM KCl, 5 mM KCl, 15 mM KCl, or 50 mM KCl. Lower salt concentrations were used for short substrates. Typical reactions contained 100 nM L1 EN enzyme in 20 μl reactions with 1X buffer 10 mM Tris pH 7.5, 5 mM MgCl, 5 mM KCl, and 100 nM DNA substrate. Reactions were typically performed for 10 to 30 min. Individual reaction conditions are noted in each experiment. For reactions measuring the rate of cutting by capillary electrophoresis, the amount of enzyme was reduced to 5 ng for mismatch DNA and 20 ng for dsDNA and time points were taken every minute from 1 to 8 min. For LC-MS analysis, 60 ng of L1 EN enzyme was incubated with 15 pmol DNA substrate with 1X buffer for 1 h in a final volume of 15 μl. Upon completion, all reactions were quenched with at least a 1:1 volume of 98% formamide and 10 mM EDTA (typically 4:1).

### Gel electrophoresis

For reactions with fluorescent substrates, a volume of quenched reaction buffer containing 0.05 to 0.1 pmol of fluorescent substrate was loaded directly onto a 10% or 15%, 7M urea denaturing PAGE gel for each reaction. To aid denaturation, substrates were usually heated at 70 °C for 5 to 10 min and the gel running buffer (1% TBE) was heated to 50 °C before being added to the gel box. 15% denaturing PAGE gels were typically run at 80 V – 150 V for 1 to 2 h. Agarose gels were run for 80 min at 80 V and stained with either ethidium bromide (Invitrogen) or SYBR Gold (Thermo Fisher Scientific). For reactions containing small nonfluorescent DNA fragments, 0.25 pmoles of DNA substrate was loaded per lane and gels were run on a 15%, 7M urea denaturing PAGE gel and stained with SYBR Gold. For reactions with plasmids or larger DNA, a volume containing at least 50 ng of plasmid was loaded onto native 1% TAE agarose or denaturing 1% TAE agarose with 1 M urea gels for each reaction or lane. For denaturing gels, DNA was ethanol precipitated from reaction buffer and resuspended in denaturing loading buffer (1 mM Tris pH 8, 8 M urea (Sigma-Aldrich), 1% (v/v) IPEGAL, 0.5 mg/ml bromophenol blue) and heat-denatured at 70 °C for 10 min as previously described (73). Native and denaturing gels were run at a constant 55 V, with denaturing TAE running buffer supplemented with 1 M urea.

### DNA substrates

DNA substrates were purchased from IDT or Genewiz (Azenta) and validated by the manufacturer using mass spectrometry. All fluorescent substrates were purchased with HPLC purification. Nonfluorescent substrates used in mass spectrometry were purchased as gel-purified oligos. All other substrates were purchased without additional purification. Plasmid containing two L1 consensus motifs was ordered from Twist Bioscience and consensus sites were removed by Q5 Site-Directed Mutagenesis (Biolabs), including recoding of internal DraI site in *ampR* gene. A complete list of DNA substrates used in this study can be found in the supplementary information.

### Sequencing

For RapidAlt sequencing, 100 nM ssDNA cut with 200 nM L1 EN (or catalytically dead enzyme) in 10 mM Tris pH 7.5, 5 mM MgCl_2_, and 5 mM KCl was hybridized to a primer at the 3′ end of the full molecule. Q5 high fidelity polymerase (NEB) was used to extend the 3′ fragment followed by ligation of the fragment to a linearized plasmid with T4 DNA ligase. Since L1 EN liberates a 5′ phosphate, kinase treatment is not needed, which helps eliminate background. PCR amplification with Q5 2X Master Mix (NEB) was performed using a primer in the plasmid and the 3′ end of the ssDNA and products were sequenced on a MiSeq at Dana Farber’s Molecular Biology Core Facilities. The procedure for sequencing double-stranded DNA was identical except that an additional primer was used for the second 3′ end to obtain coverage for both strands. This procedure is sufficient to detect all cuts downstream of the primer excluding only short cuts that might be made in the primer-binding region.

### Endonuclease cleavage analysis by liquid chromatography-tandem mass spectrometry

Endonuclease cleavage reactions were performed as described above. DNA was purified using Oligo Clean and Concentrator columns (Zymo Research) and eluted with 16 μl nuclease free water. Eluted samples were filtered using Durapore PVDF 0.22 μm MC-GV spin filters. Liquid chromatography-tandem mass spectrometry analysis was performed on a Vanquish Horizon UHPLC System equipped with a diode array detector and a Thermo Q-Exactive Plus mass spectrometer operating under negative electrospray ionization mode (–ESI). UHPLC was performed using a Waters ACQUITY Premier Oligonucleotide C18 Column (2.1′ 100 mm, 1.7 μm) at 70 °C and 0.4 ml/min flow rate, with a gradient mobile phase consisting of methanol and N,N-Diisopropylethylamine-1,1,1,3,3,3-hexafluoro-2-propanol (DIEA-HFIP) ion-paring aqueous buffer. UV signal was recorded at 260 nm. MS data acquisition was performed in the scan mode at 70,000 mass resolution. MS raw data were deconvoluted using BioPharma Finder 4.1 (Thermo Fisher Scientific).

### DNA structural analysis

DNA structures were obtained from the UNAfold DNA Folding Form (http://www.unafold.org/mfold/applications/dna-folding-form.php) using default parameters with 50 mM Na^+^ and 5 mM Mg^2+^ and visualized with forna (http://rna.tbi.univie.ac.at/forna/) and Adobe Illustrator version 28.3.

### Gel quantification and graph generation

Gels were quantified using ImageJ version 1.54 g and visualized in Graphpad Prism version 10.4.0.

## Data availability

All data are available either in this article or in the supplementary information. Requests for further information about the data may be made to the corresponding authors.

## Supporting information

This article contains supporting information.

## Conflict of interest

M. S. T. has received consulting fees from ROME Therapeutics and Tessera Therapeutics that are not related to this work. M. S. T. and J. L. have equity in ROME therapeutics. K. H. B. declares relationships with Alamar Biosciences, Genscript, Oncolinea/PrimeFour Therapeutics, ROME Therapeutics, Scaffold Therapeutics, Tessera Therapeutics, and Transposon Therapeutics. The other authors declare that they have no conflicts of interest with the contents of this article
