# Supplementary material for: Rapid DNA cleavage by the LINE-1 endonuclease proximal to DNA ends and at mismatches
Source: J Biol Chem. 2025 Nov 29;302(1):110994. doi: 10.1016/j.jbc.2025.110994 (PMC12809410; doi:10.1016/j.jbc.2025.110994)
Supplement: Supplementary Material 1 [file mmc1.docx]

**Supplementary Information for:**

**Rapid DNA Cleavage by the LINE-1 Endonuclease at Mismatches and Proximal to DNA Ends
Bryant D. Miller, Benedict A. Smail et al.**

Correspondence :  [jlacava@rockefeller.edu](mailto:jlacava@rockefeller.edu); [kathleenh_burns@dfci.harvard.edu](mailto:kathleenh_burns@dfci.harvard.edu); [martin_taylor@brown.edu](mailto:martin_taylor@brown.edu)

Table of Contents

[Supplementary Figures 2](#_Toc213192996)

[Supplementary Figure 1: Validation of L1 EN catalytically dead mutants. 2](#_Toc213192997)

[Supplementary Figure 2: L1 EN cutting in varying concentrations of KCl. 3](#_Toc213192998)

[Supplementary Figure 3: APE1 and L1 EN cutting. 4](#_Toc213192999)

[Supplementary Figure 4: Quantification of RapidAlt vs. MinHP. 5](#_Toc213193000)

[Supplementary Figure 5: Gels quantified in Figure 3D. 6](#_Toc213193001)

[Supplementary Figure 6: Gels quantified in Figure 4A. 7](#_Toc213193002)

[Supplementary Figure 7: Testing a secondary structure consensus structure. 8](#_Toc213193003)

[Supplementary Figure 8: Predicted Structure for 90mer “ssDNA bottom” 9](#_Toc213193004)

[Supplementary Figure 9: Cy5 channel quantified in Figure 6A. 10](#_Toc213193005)

[Supplementary Figure 10: Gels Quantified in Figure 7 D and E. 11](#_Toc213193006)

[Supplementary Figure 11: Gels used to quantify overhang sequence variation in Figure 8A. 12](#_Toc213193007)

[Supplementary Figure 12: Gels and quantifications for Figure 8B. 13](#_Toc213193008)

[Supplementary Figure 13: L1 EN cutting – 5’ vs no overhang. 14](#_Toc213193009)

[Supplementary Figure 14: Gels quantified to generate the percent substrate cleaved in Figure 8C. 15](#_Toc213193010)

[Supplementary Tables 16](#_Toc213193011)

[Table S1: Enzymes, Substrates, and Plasmids Used in This Study 16](#_Toc213193012)

[Table S2: Sequencing results 21](#_Toc213193013)

[Table S3: Dinucleotide frequencies at L1 insertion breakpoints 24](#_Toc213193014)

# Supplementary Figures


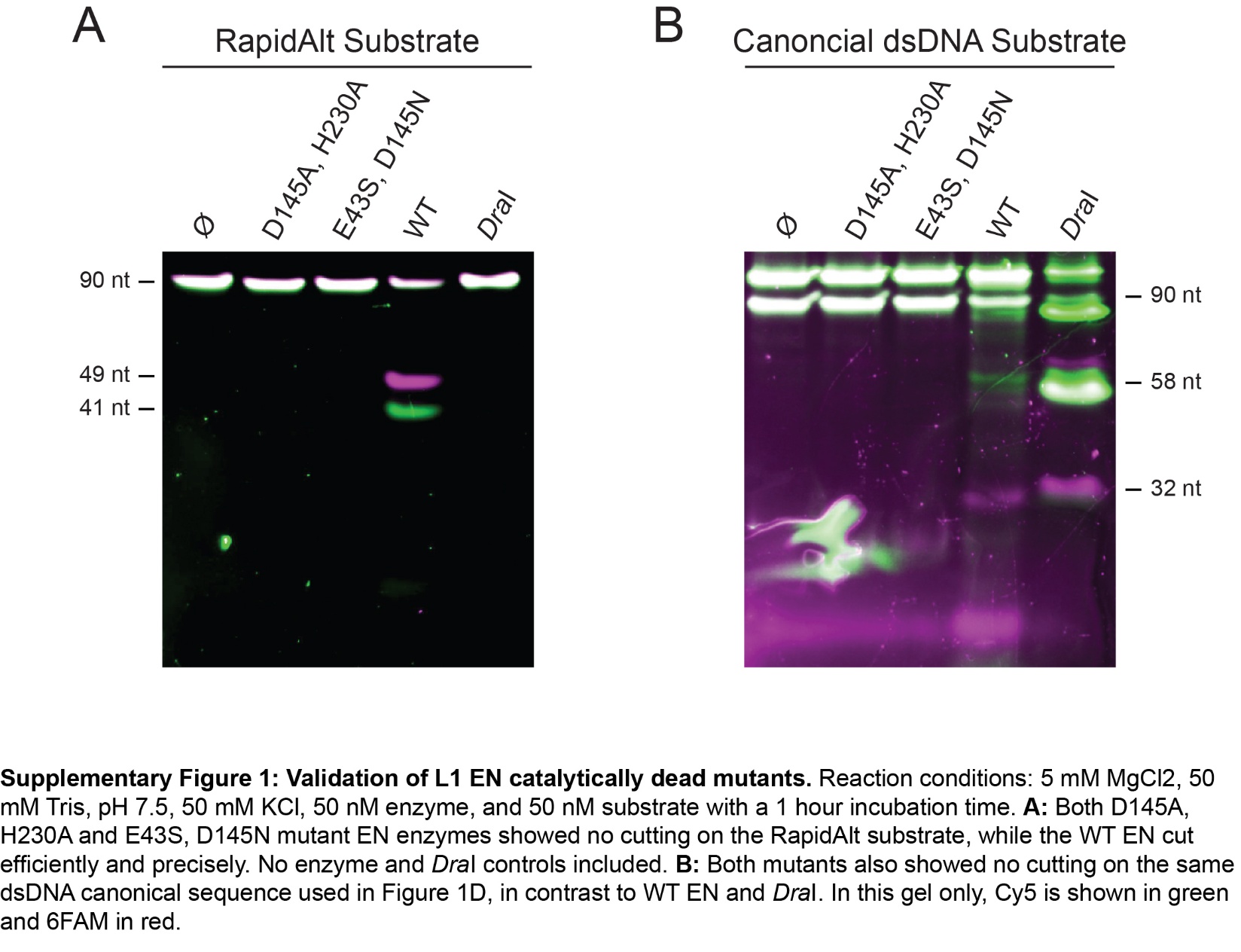


Supplementary Figure 1: Validation of L1 EN catalytically dead mutants. Reaction conditions: 5 mM MgCl2, 50 mM Tris, pH 7.5, 50 mM KCl, 50 nM enzyme, and 50 nM substrate with a 1 hour incubation time and the indicated added enzymes; Ø: no enzyme added. **A:** Both D145A, H230A and E43S, D145N mutant EN enzymes showed no cutting on the RapidAlt substrate, while the WT EN cut efficiently and precisely. *Dra*I and no-enzyme and controls included. **B:** Both mutants also showed no cutting on the same dsDNA canonical sequence used in Figure 1D, in contrast to WT EN and *Dra*I. In this gel only, Cy5 is shown in green and 6FAM in red.


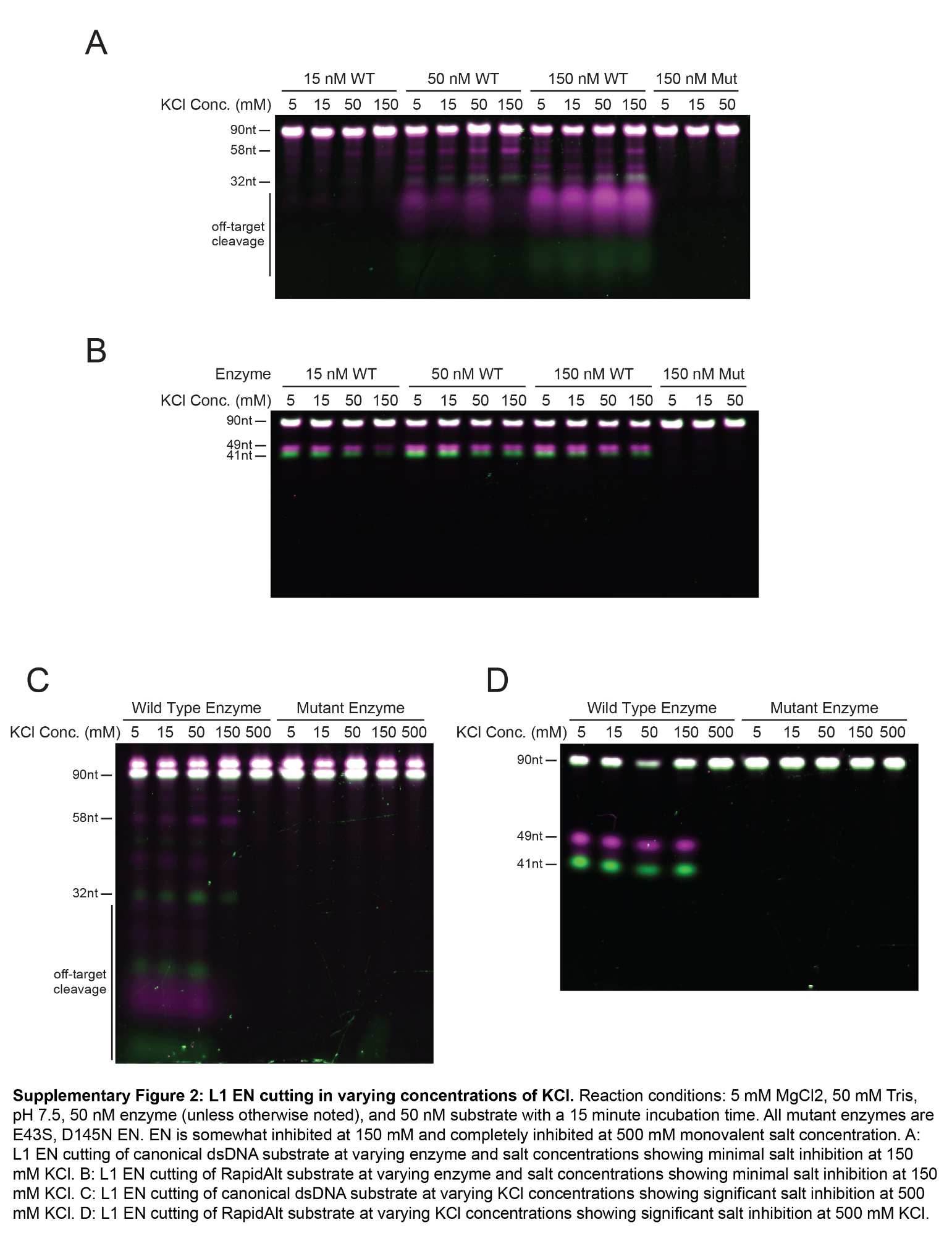


Supplementary Figure 2: L1 EN cutting in varying concentrations of KCl. Reaction conditions: 5 mM MgCl2, 50 mM Tris, pH 7.5, 50 nM enzyme (unless otherwise noted), and 50 nM substrate with a 15 minute incubation time. All mutant enzymes are E43S, D145N EN. EN is somewhat inhibited at 150 mM and completely inhibited at 500 mM monovalent salt concentration. **A:** L1 EN cutting of canonical dsDNA substrate at varying enzyme and salt concentrations showing minimal salt inhibition at 150 mM KCl. **B:** L1 EN cutting of RapidAlt substrate at varying enzyme and salt concentrations showing minimal salt inhibition at 150 mM KCl. **C:** L1 EN cutting of canonical dsDNA substrate at varying KCl concentrations showing significant salt inhibition at 500 mM KCl. **D:** L1 EN cutting of RapidAlt substrate at varying KCl concentrations showing significant salt inhibition at 500 mM KCl.


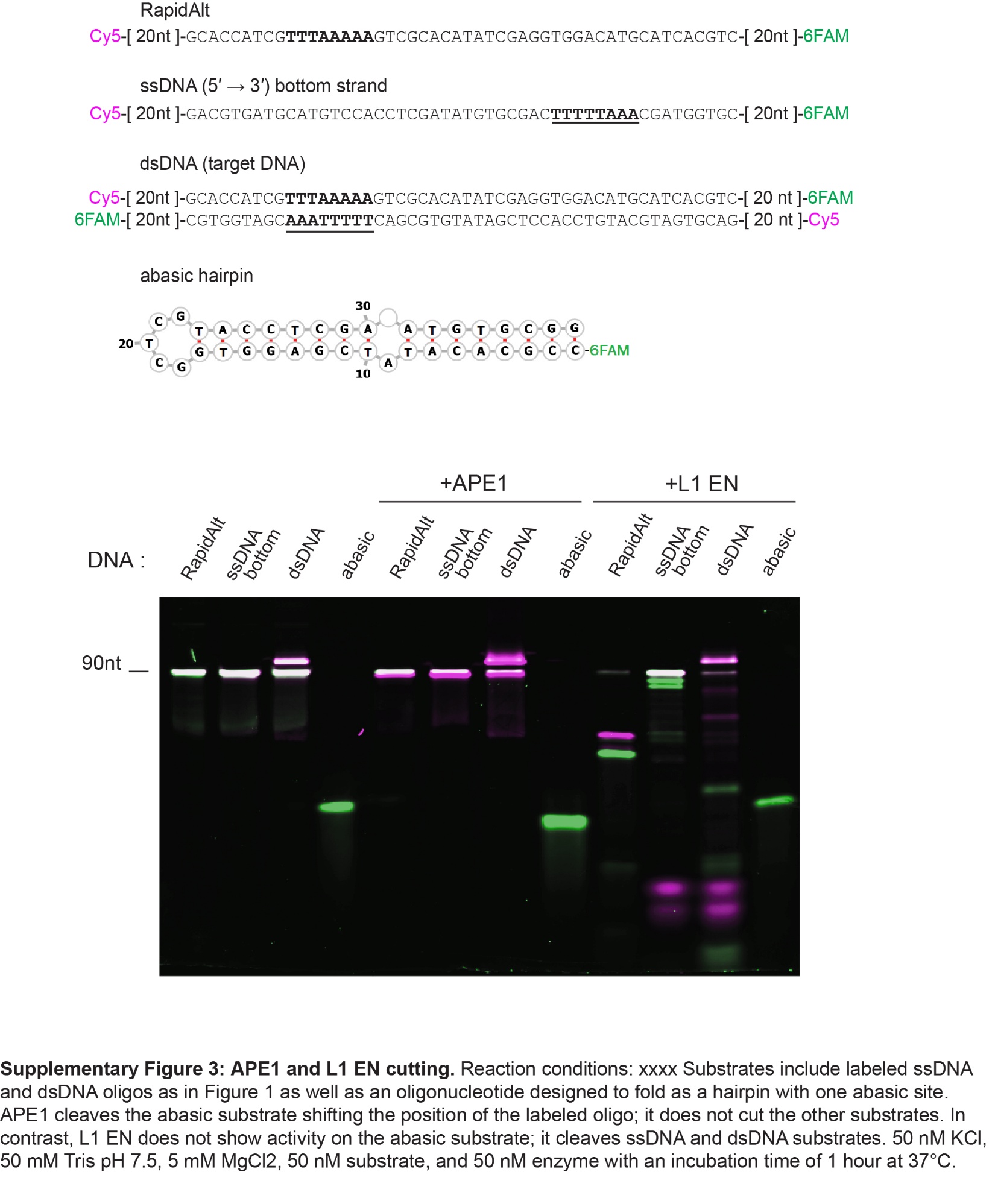


Supplementary Figure 3: APE1 and L1 EN cutting. Substrates include the same labeled oligos as in Figure 1 (RapidAlt, ssDNA bottom, and dsDNA including the canonical cut site) as well as an oligonucleotide designed to fold as a hairpin with one abasic site. APE1 cleaves the abasic substrate shifting the position of the labeled oligo on the gel; it does not cut the other substrates. In contrast, L1 EN does not show activity on the abasic substrate but cleaves RapidAlt, ssDNA bottom, and dsDNA substrates. Reaction conditions: 50 mM KCl, 5 mM MgCl2, 50 mM Tris pH 7.5, 50 nM enzyme, and 50 nM substrate in 50 μL for 1 hour at 37°C.


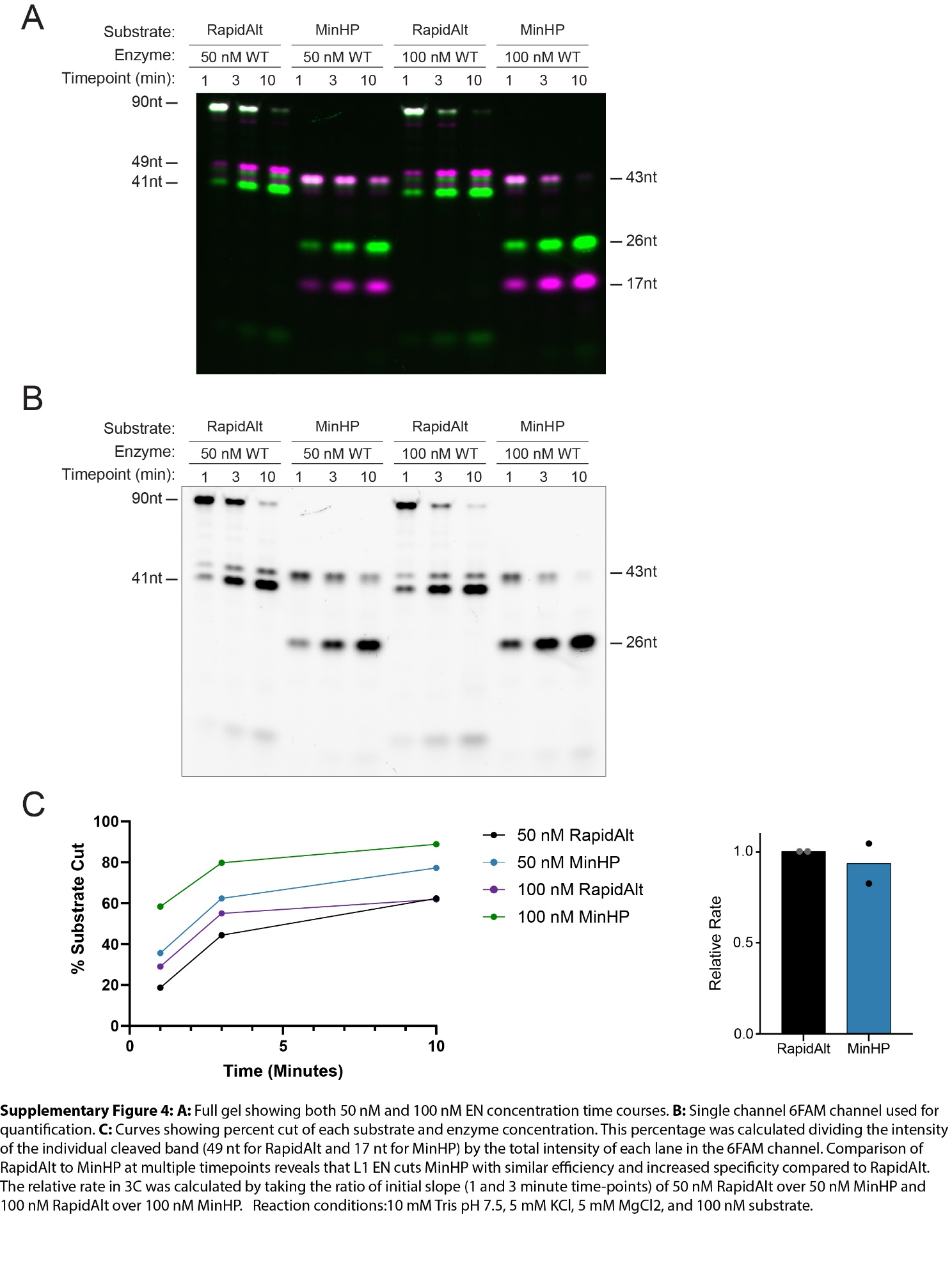


Supplementary Figure 4: Quantification of RapidAlt vs. MinHP. **A:** Full gel showing both 50 nM and 100 nM EN concentration time courses. B: Single channel 6FAM channel used for quantification. C: Curves showing percent cut of each substrate and enzyme concentration. This percentage was calculated dividing the intensity of the individual cleaved band (49 nt for RapidAlt and 17 nt for MinHP) by the total intensity of each lane in the 6FAM channel. Comparison of RapidAlt to MinHP at multiple timepoints reveals that L1 EN cuts MinHP with similar efficiency and increased specificity compared to RapidAlt. The relative rate in 3C was calculated by taking the ratio of initial slope (1 and 3 minute time-points) of 50 nM RapidAlt over 50 nM MinHP and 100 nM RapidAlt over 100 nM MinHP. Reaction conditions:10 mM Tris pH 7.5, 5 mM KCl, 5 mM MgCl2, and 100 nM substrate.


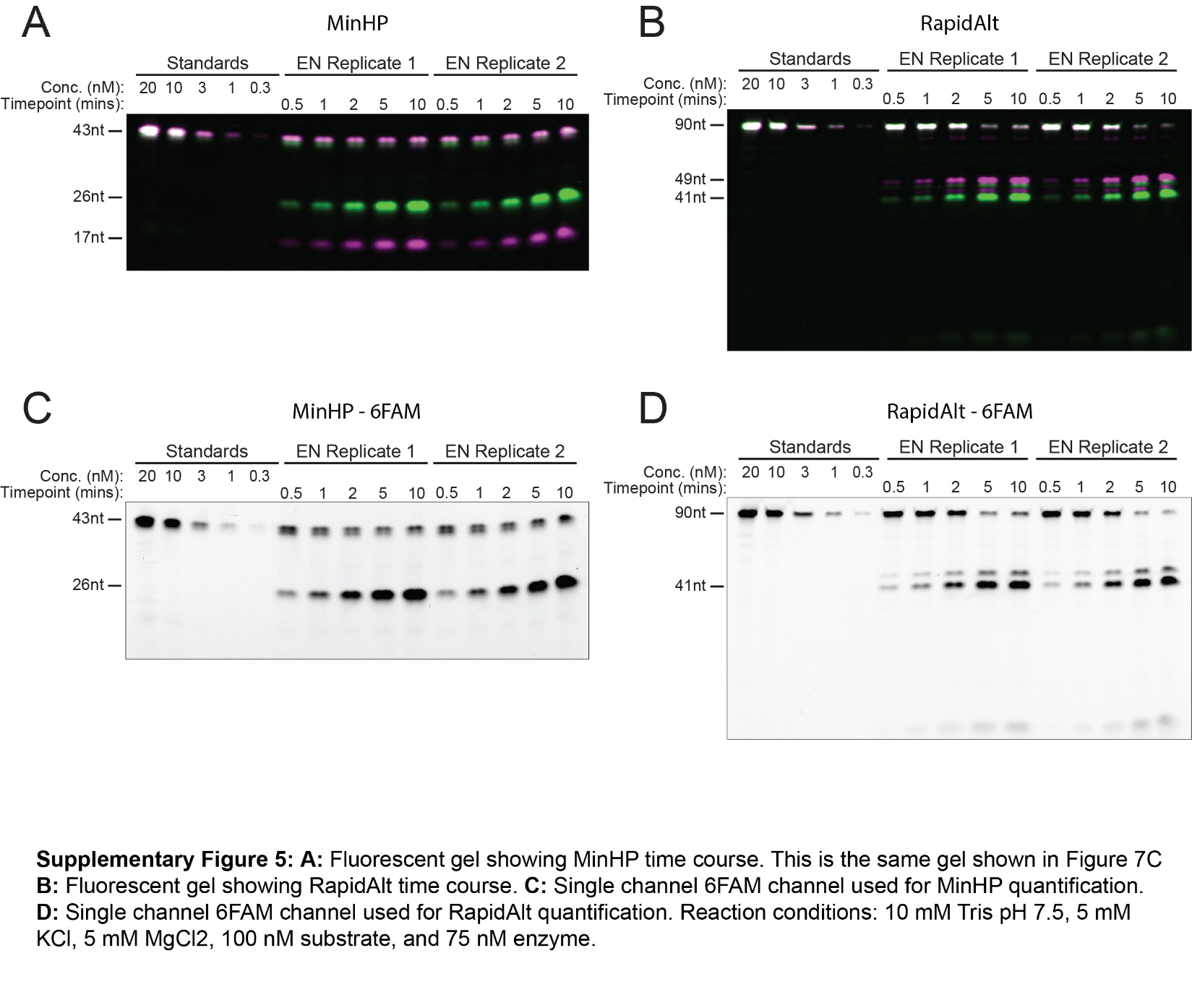


Supplementary Figure 5: Gels quantified in Figure 3D. **A:** Fluorescent gel showing MinHP time course. This is the same gel shown in Figure 7C and Supplementary Figure 10A. **B:** Fluorescent gel showing RapidAlt time course. **C:** Single channel 6FAM channel used for MinHP quantification. **D:** Single channel 6FAM channel used for RapidAlt quantification. Reaction conditions: 10 mM Tris pH 7.5, 5 mM KCl, 5 mM MgCl2, 100 nM substrate, and 75 nM enzyme, 37°C.


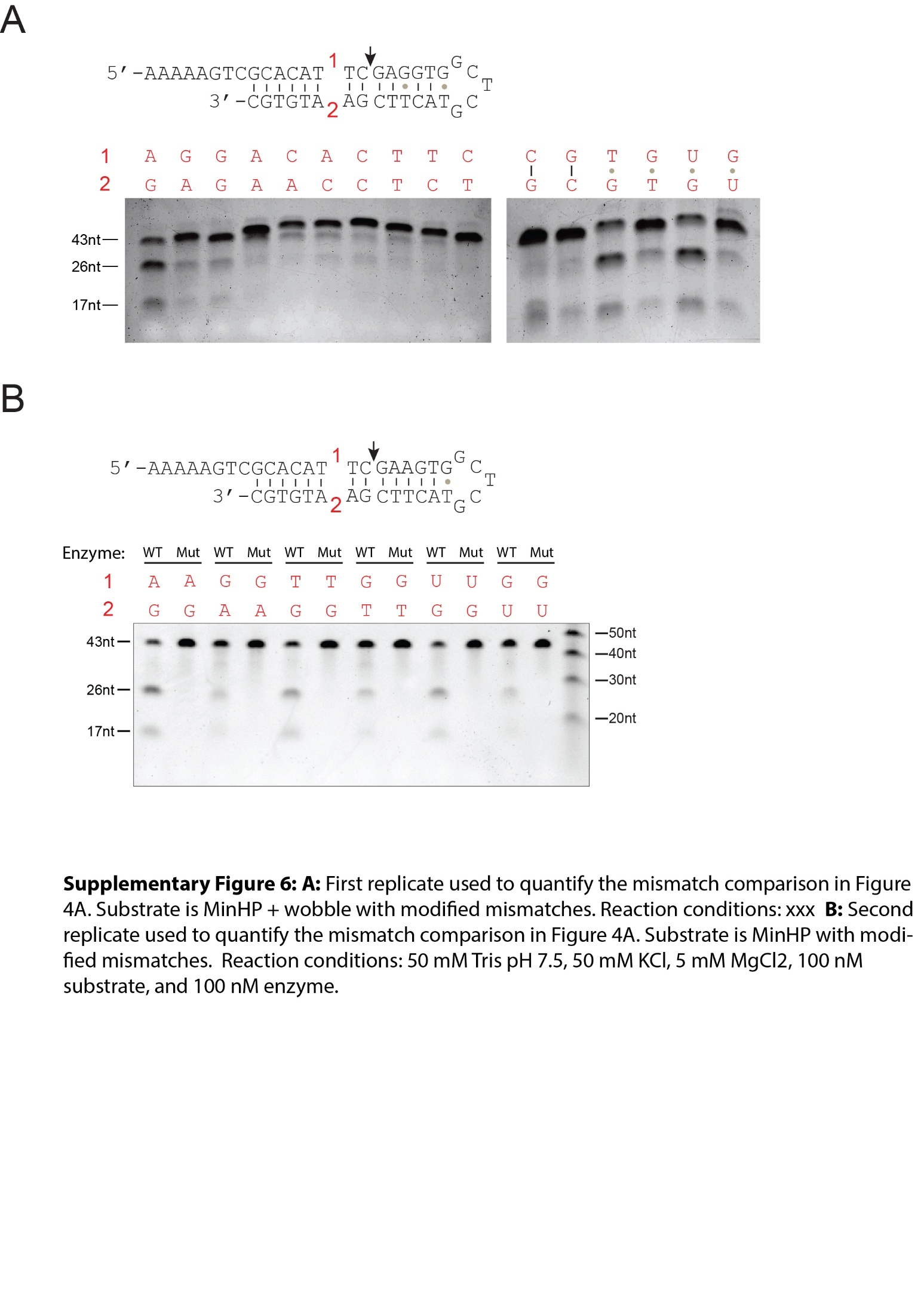


Supplementary Figure 6: Gels quantified in Figure 4A. **A:** First replicate used to quantify the mismatch comparison in Figure 4A. Substrate is MinHP + wobble with modified mismatches. Reaction conditions: 50 mM Tris pH 7.5, 50 mM KCl, 5 mM MgCl2, 100 nM substrate, and 100 nM enzyme with an incubation time of 10 minutes at 37°C. **B:** Second replicate used to quantify the mismatch comparison in Figure 4A. Substrate is MinHP with modified mismatches. Reaction conditions: 5 mM MgCl2, 50 mM Tris pH 7.5, 50 nM enzyme, and 50 nM substrate in 50 μL for 1 hour at 37°C.


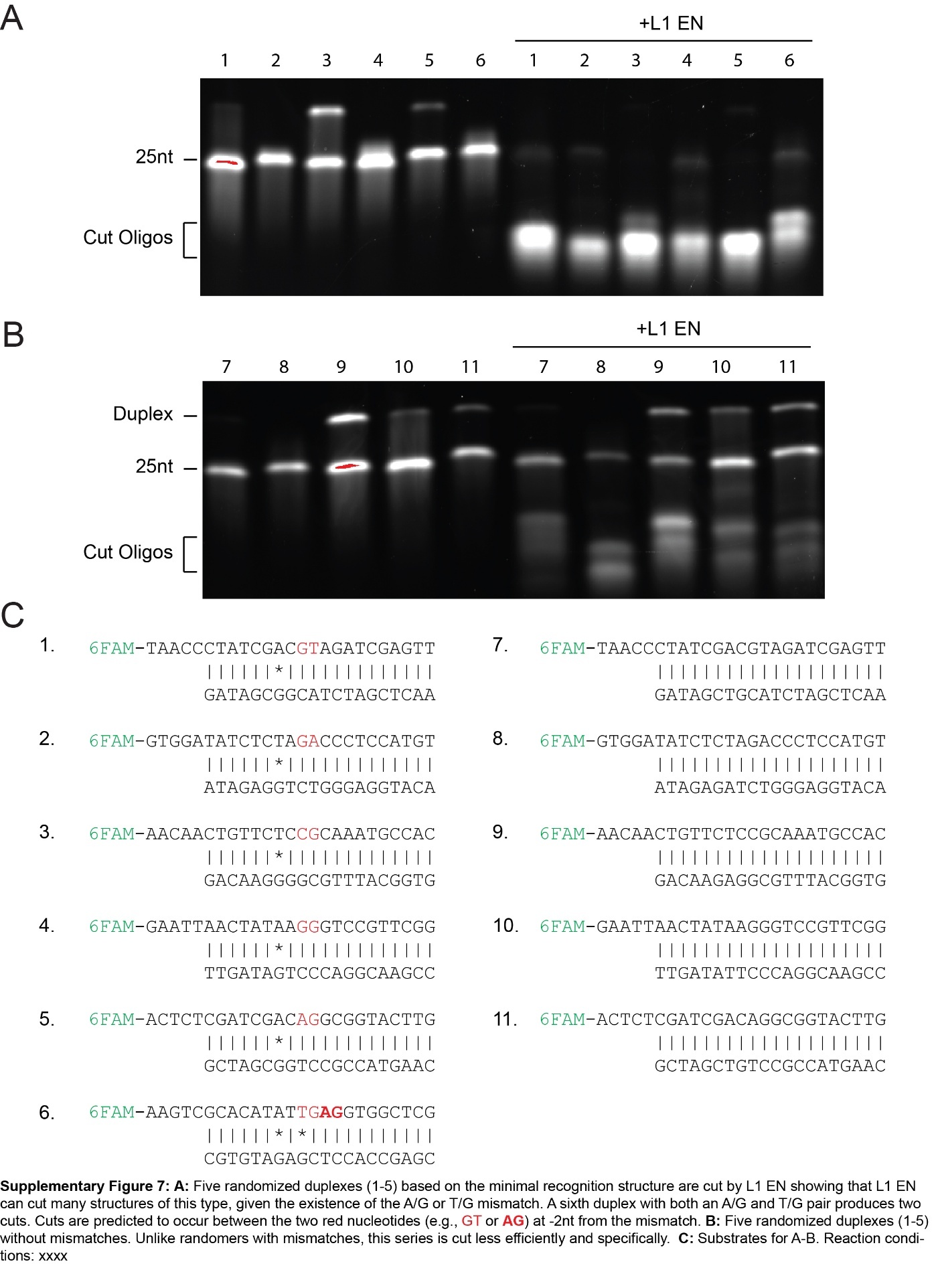


Supplementary Figure 7: Testing a secondary structure consensus structure. Reaction conditions: 5 mM MgCl2, 50 mM Tris pH 7.5, 50 nM enzyme, and 50 nM substrate in 50 μL for 1 hour at 37°C. **A:** Five randomized duplexes (1-5) based on the minimal recognition structure are cut by L1 EN showing that L1 EN can cut many structures of this type, given the existence of the A/G or T/G mismatch. A sixth duplex with both an A/G and T/G pair produces two cuts. Cuts are predicted to occur between the two red nucleotides (e.g., GT or AG) at -2nt from the mismatch. **B:** Five randomized duplexes (1-5) without mismatches. Unlike randomers with mismatches, this series is cut less efficiently and specifically.  **C:** Substrates for A and B.

**
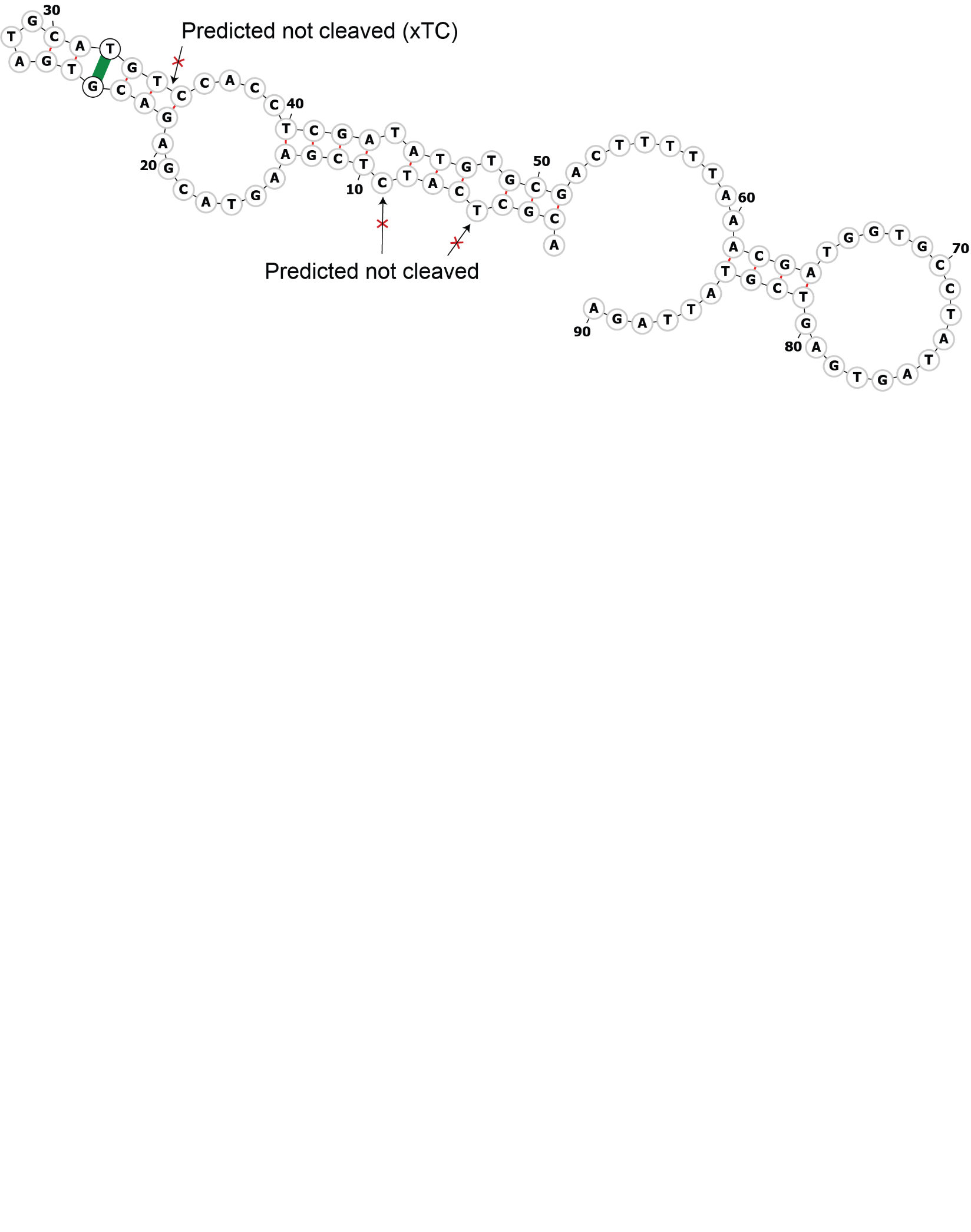
**

Supplementary Figure 8: Predicted Structure for 90mer “ssDNA bottom” does not contain mismatches predicted to be cut by L1 EN. DNA secondary structure was obtained from the UNAfold DNA Folding Form using default parameters with 50 mM Na+ and 5 mM Mg2+, visualized with forna, and edited with Adobe Illustrator for clarity. C-T and T-T mismatches were not cleaved in our data; note that a small amount of inefficient cleavage induced by these mismatches could explain removal of a few bases from the 5’ end and the ‘green’ bands just under 90nt in “ssDNA bottom” substrate (**Figure 1D)**. Cleavage after the G-T wobble would occur two bases downstream of the T (GT↓C), as indicated, but cleavage is not predicted because the xT↓C sequence strongly inhibited cleavage in our experiments (see **Figure 4B**).


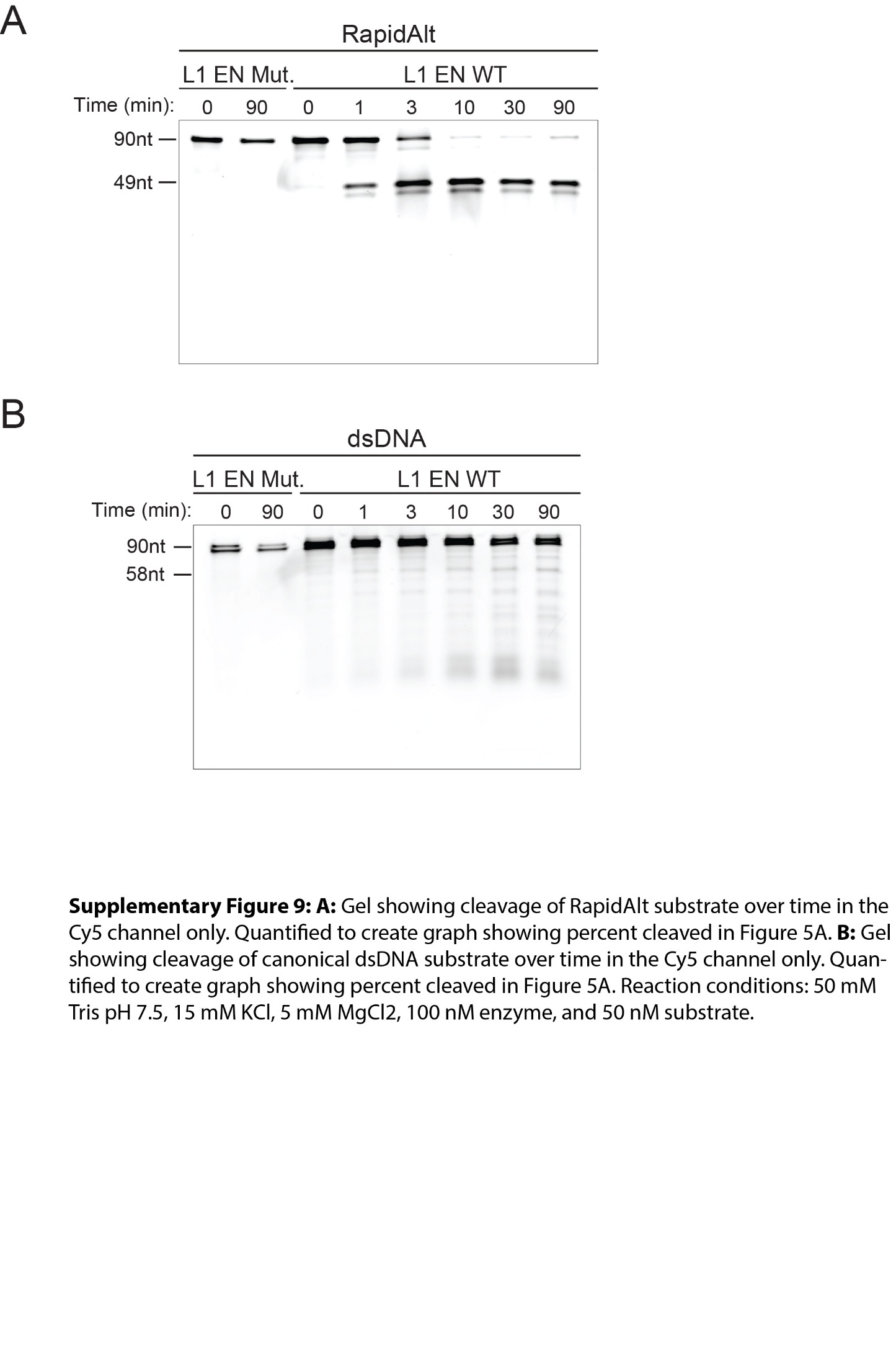


Supplementary Figure 9: Cy5 channel quantified in Figure 6A. **A:** Gel showing cleavage of RapidAlt substrate over time in the Cy5 channel only. Quantified to create graph showing percent cleaved in Figure 6A. **B:** Gel showing cleavage of canonical dsDNA substrate over time in the Cy5 channel only. Quantified to create graph showing percent cleaved in Figure 6A. Reaction conditions: 50 mM Tris pH 7.5, 15 mM KCl, 5 mM MgCl2, 100 nM enzyme, and 50 nM substrate, 37°C.

**
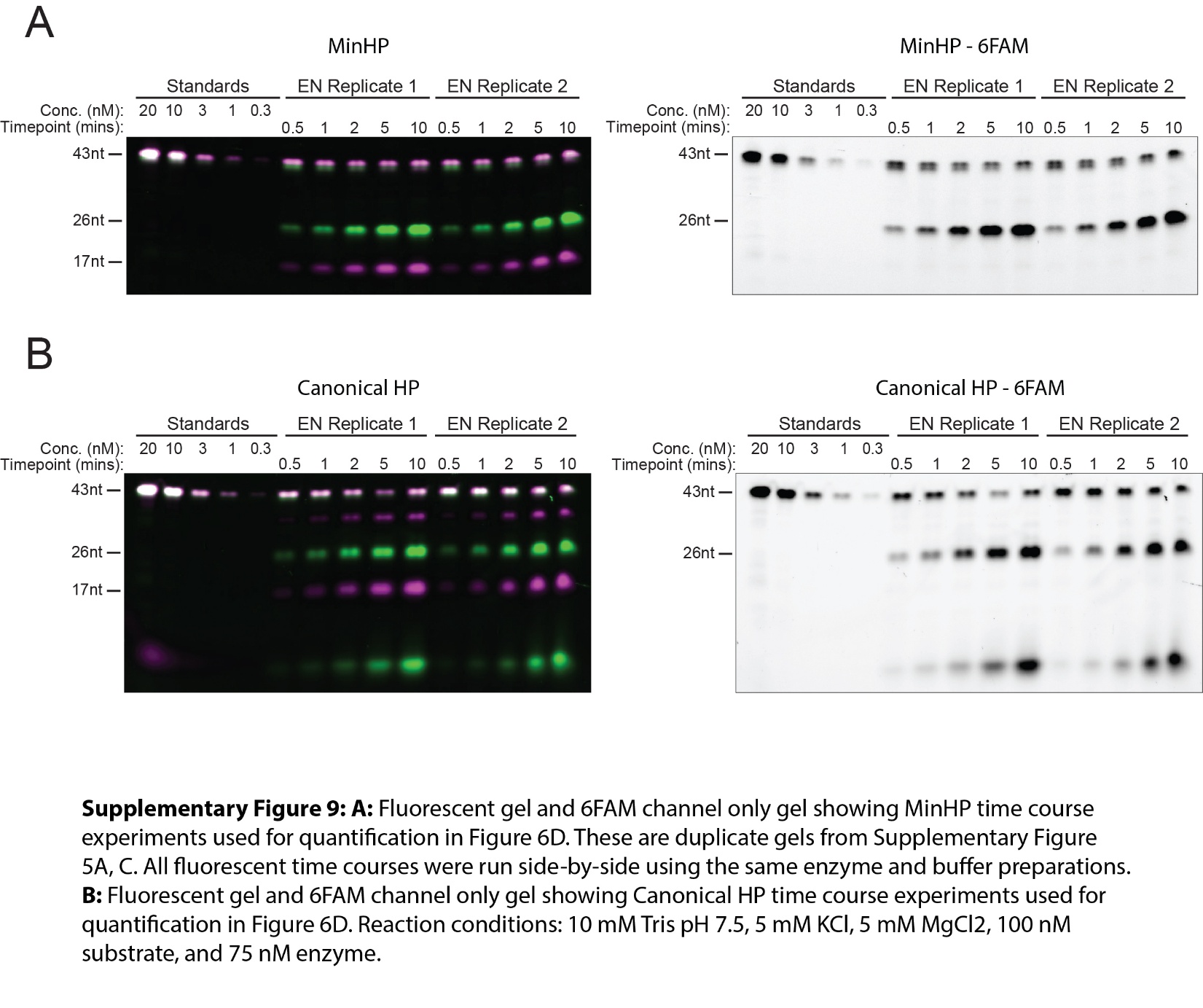
**

Supplementary Figure 10: Gels Quantified in Figure 7D and E. **A:** Fluorescent gel and 6FAM channel only gel showing MinHP time course experiments used for quantification in Figure 7D and E. These are duplicate images from Figure 7C and Supplementary Figure 5A, C. All fluorescent time courses were run side-by-side using the same enzyme and buffer preparations. **B:** Fluorescent gel and 6FAM channel only gel showing Canonical HP time course experiments used for quantification in Figure 7D. Reaction conditions: 10 mM Tris pH 7.5, 5 mM KCl, 5 mM MgCl2, 100 nM substrate, and 75 nM enzyme, 37°C.


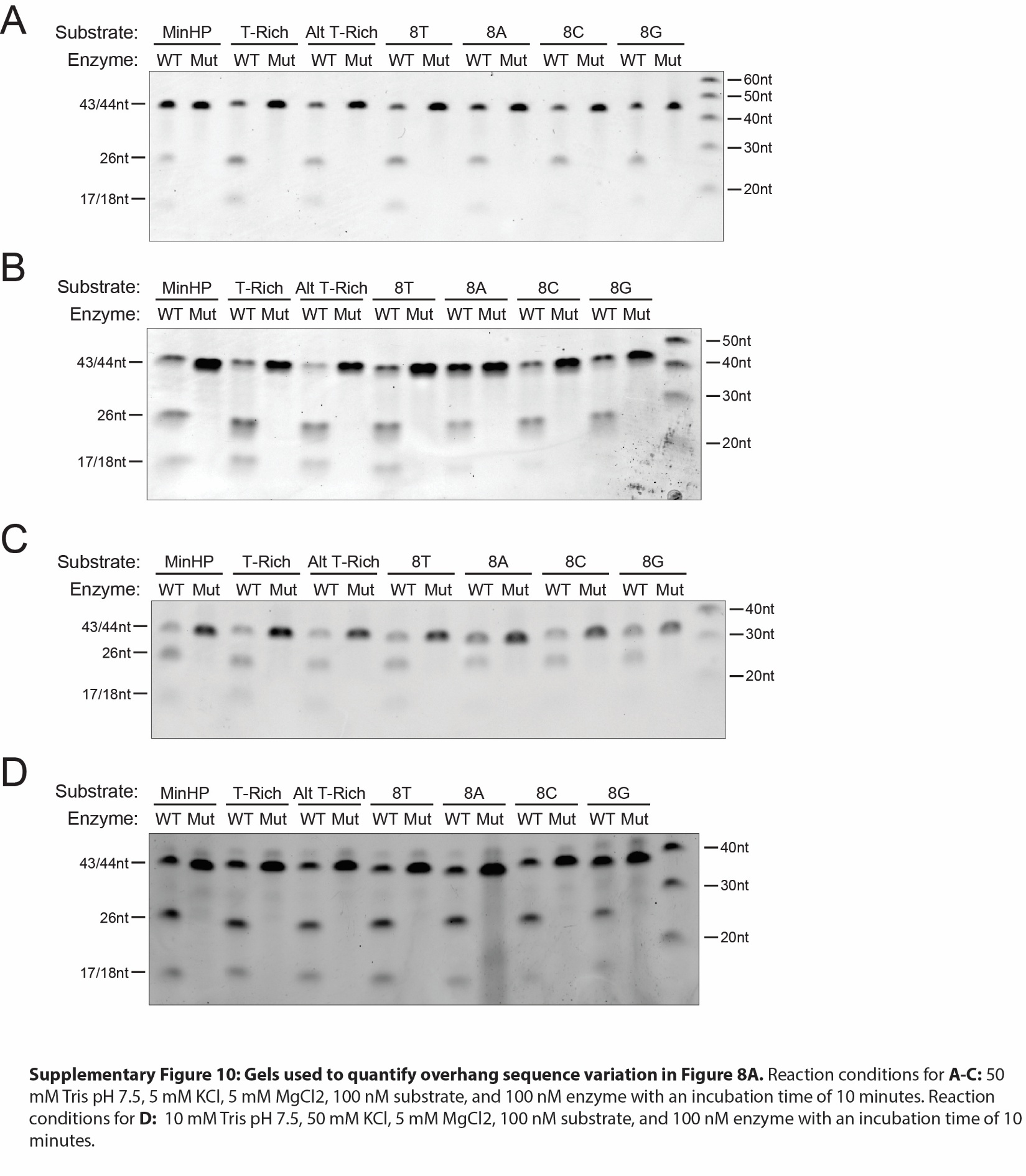


Supplementary Figure 11: Gels used to quantify overhang sequence variation in Figure 8A. Reaction conditions for **A-C:** 50 mM Tris pH 7.5, 5 mM KCl, 5 mM MgCl2, 100 nM substrate, and 100 nM enzyme with an incubation time of 10 minutes at 37°C. Reaction conditions for **D:** 10 mM Tris pH 7.5, 50 mM KCl, 5 mM MgCl2, 100 nM substrate, and 100 nM enzyme with an incubation time of 10 minutes at 37°C.

**
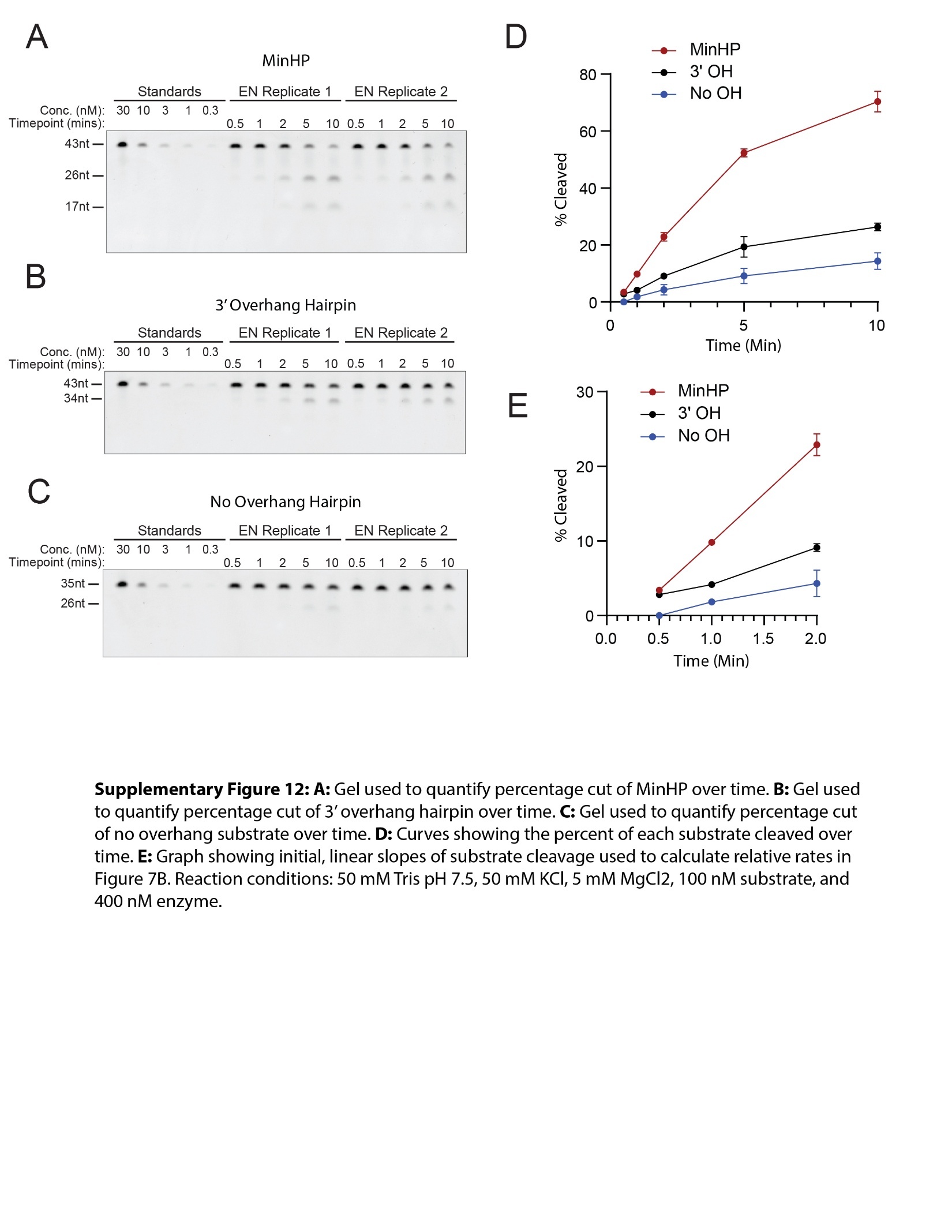
**

Supplementary Figure 12: Gels and quantifications for Figure 8B. **A:** Gel used to quantify percentage cut of MinHP over time. **B:** Gel used to quantify percentage cut of 3’ overhang hairpin over time. **C:** Gel used to quantify percentage cut of no overhang substrate over time. **D:** Curves showing the percentage of each substrate cleaved over time. **E:** Graph showing initial, linear slopes of substrate cleavage used to calculate relative rates in Figure 8B. Reaction conditions: 50 mM Tris pH 7.5, 50 mM KCl, 5 mM MgCl2, 100 nM substrate, and 400 nM enzyme at 37°C.


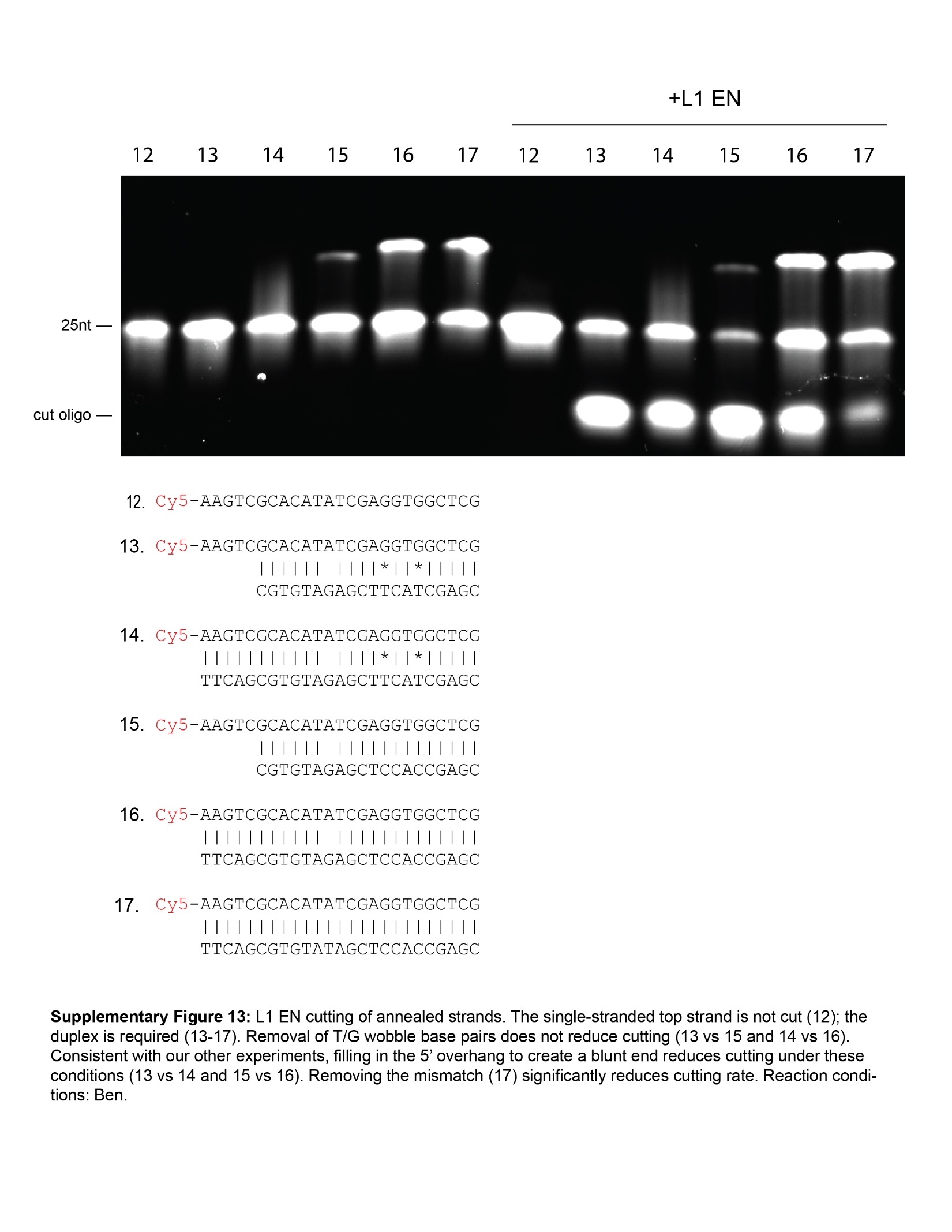


Supplementary Figure 13: L1 EN cutting – 5’ vs no overhang. The single-stranded top strand is not cut (12); the duplex is required (13-17). Removal of T/G wobble base pairs does not reduce cutting (13 vs 15 and 14 vs 16). Consistent with our other experiments, filling in the 5’ overhang to create a blunt end reduces cutting under these conditions (13 vs 14 and 15 vs 16). Removing the mismatch (17) significantly reduces cutting rate. Reaction conditions: 5 mM MgCl2, 50 mM Tris pH 7.5, 50 nM enzyme, and 50 nM substrate in 50 μL for 1 hour at 37°C.

t


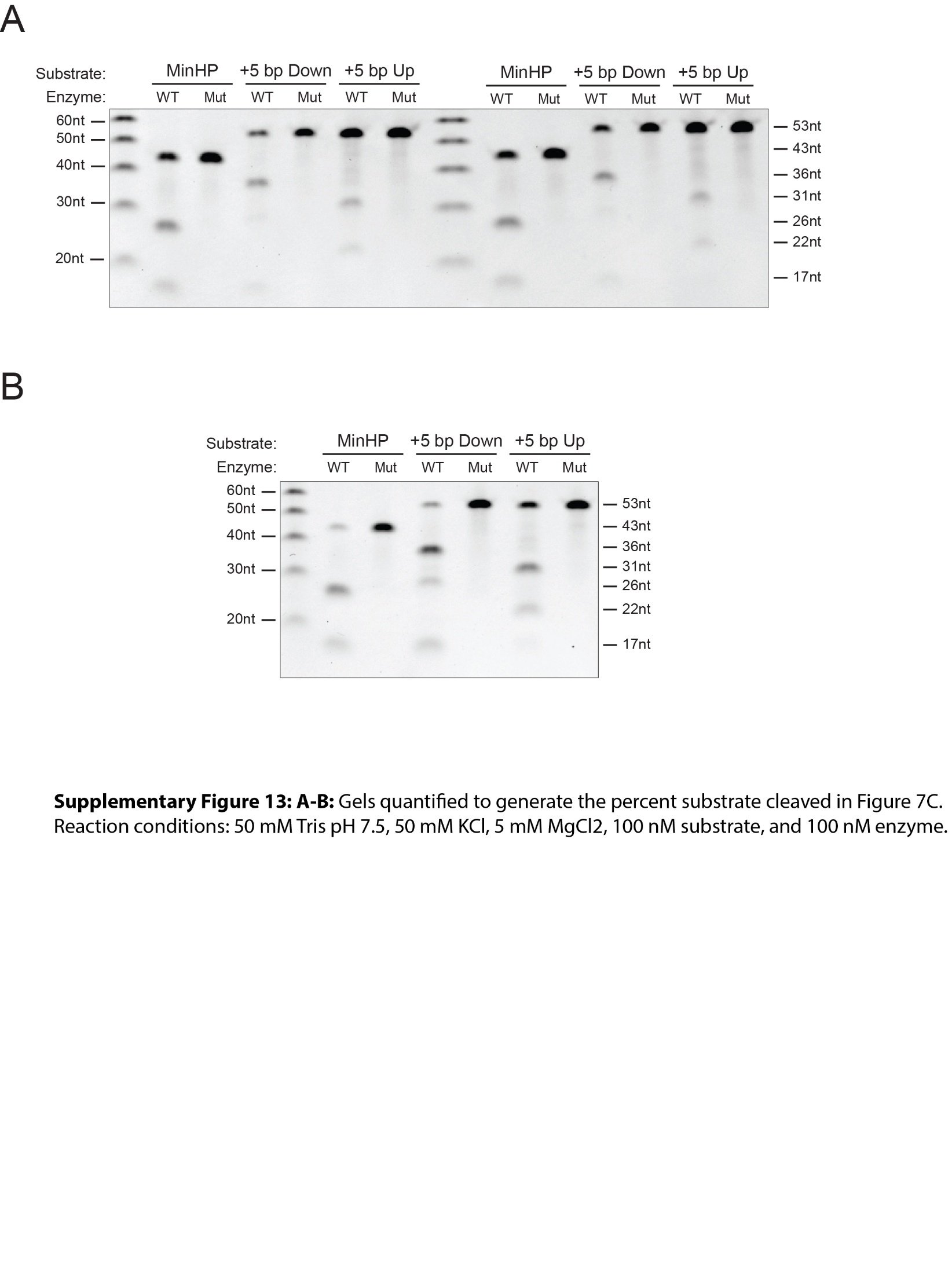


Supplementary Figure 14: Gels quantified to generate the percent substrate cleaved in Figure 8C. Reaction conditions: 50 mM Tris pH 7.5, 50 mM KCl, 5 mM MgCl2, 100 nM substrate, and 100 nM enzyme with a 10-minute incubation time.

# Supplementary Tables

## Table S1: Enzymes, Substrates, and Plasmids Used in This Study

All plasmids have been deposited in Addgene.

| **Name:** | **Sequence / Description:** | **Purpose and Figure(s):** |
| --- | --- | --- |
| pMT924 | His10-SUMO-Orf2 EN WT (1-238) in pFloat-T7 (bacterial expression) | WT EN expression |
| pMT926 | His10-SUMO-Orf2 EN (1-238, D145A + H230A) in pFloat-T7 | Catalytic mutant EN |
| pMT1117 | His10-SUMO-Orf2 EN (E43S + D145N) in pFloat-T7 (‘Mutant EN’ and “Mut EN” used in much of the paper) | Catalytic mutant EN |
| pMT1207, Plasmid with 2 DraI sites | AAAAAAACCACCGCTACCAGCGGTGGTTTGTTTGCCGGATCAAGAGCTACCAACTCTTTTTCCGAGGTAACTGGCTTCAGCAGAGCGCAGATACCAAATACTGTTCTTCTAGTGTAGCCGTAGTTAGGCCACCACTTCAAGAACTCTGTAGCACCGCCTACATACCTCGCTCTGCTAATCCTGTTACCAGTGGCTGCTGCCAGTGGCGATAAGTCGTGTCTTACCGGGTTGGACTCAAGACGATAGTTACCGGATAAGGCGCAGCGGTCGGGCTGAACGGGGGGTTCGTGCACACAGCCCAGCTTGGAGCGAACGACCTACACCGAACTGAGATACCTACAGCGTGAGCTATGAGAAAGCGCCACGCTTCCCGAAGGGAGAAAGGCGGACAGGTATCCGGTAAGCGGCAGGGTCGGAACAGGAGAGCGCACGAGGGAGCTTCCAGGGGGAAACGCCTGGTATCTTTATAGTCCTGTCGGGTTTCGCCACCTCTGACTTGAGCATCGATTTTTGTGATGCTCGTCAGGGGGGCGGAGCCTATGGAAAAACGCCAGCAACGCAGAAAGGCCCACCCGAAGGTGAGCCAGGTGATTACATTTAGGTCCTCAAGAAAGGCCCACCCGAAGGTGAGCCAGGTGATTACATTTAGGTCCTCAAGAAAGGCCCACCCGAAGGTGAGCCAGGTGATTACATTTAGGTCCTCAAGAAAGGCCCACCCGAAGGTGAGCCAGGTGATTACATTTAGGTCCTCAAGAAAGGCCCACCCGAAGGTGAGCCAGGTGATTACATTTGGGCCCTCATTACCAATGCTTAATCAGTGAGGCACCTATCTCAGCGATCTGTCTATTTCGTTCATCCATAGTTGCCTGACTCCCCGTCGTGTAGATAACTACGATGCGGGAGGGCTTACCATCTGGCCCCAGTGCTGCAATGATACCGCGAGACCCACGCTCACCGGCTCCAGATTTATCAGCAATAAACCAGCCAGCCGGGAGGGCCGAGCGCAGAAGTGATCCTGCAACTTTATCCGCCTCCATCCAGTCTATTAATTGTTGCCGGGAAGCTAGAGTAAGTAGTTCGCCAGTTAATAGTTTGCGCAACGTTGTTGCCATTGCTACAGGCATCGTGGTGTCACGCTCGTCGTTTGGTATGGCTTCATTCAGCTCCGGTTCCCAACGATCAAGGCGAGTTACATGATCCCCCATGTTGTGCAAAAAAGCGGTTAGCTCCTTCGGTCCTCCGATCGTTGCCAGAAGTAAGTTGGCCGCAGTGTTATCACTCATGGTTATGGCAGCACTGCATAATTCTCTTACTGTCATGCCATCCGTGAGATGCTTTTCTGTGACTGGTGAGTACTCAACCAAGTCATTCTGAGAATAGTGTATGCGGCGACCGAGTTGCTCTTGCCCGGCGTCAATACGGGATAATACCGCGCCACATAGCAGAACTTTAAAAGTGCTCATCATTGGAAAACGTTCTTCGGGGCGTAAACTCTCAAGGATCTTACCGCTGTTGAGATCCAGTTCGATGTAACCCACTCGTGCACCCAACTGATCTTCAGCATCTTTTACTTTCACCAGCGTTTCTGGGTGAGCAAAAACAGGAAGGCAAAATGCCGCAAAAAAGGGAATAAGGGCGACACGGAAATGTTGAATACTCATTTTAGCTTCCTTAGCTCCTGAAAATCTCGATAACTCAAAAAATACGCCCGGTAGTGATCTTATTTCATTATGGTGAAAGTTGGAACCTCTTACGTGCCGATCAAGTCAAAAGCCTCCGGTCGGAGGCTTTTGACTTTCTGCTATGGAGGTCAGGTATGATTTAAATGGTCAGTATTGAGCCTCAGGAAACAGCTATGACATCAAGCTGACTAGATAATCTAGCTGATCGTGGACCGATCATACGTATAATGCCGTAAGATCACGCGCCTTCTCCCTTAGCCTACCGAAGTAGCCCAGGTCGGACCGCGAGGAGGTGGAGATGCCATGCCGACCCTGTCATGGATACGATCGTACTCGGAACCTATAGTGAGTCGTATTAGAGGCCGCATATCATTCAGGACGAGCCTCAGACTCCAGCGTAACTGGACTGCAATCAACTCACTGGCTCACCTTCCGGTCCACGATCAGCTAGAATCAAGCTGACTAGATAAACTGGCCGTCGTTTTACACGGGTGGGCCTTTCTTCGGTAGAAAATCAAAGGATCTTCTTGAGATCCTTTTTTTCTGCGCGTAATCTGCTGCTTGCAAAC | Fig 1C |
| pMT1208, Plasmid with 1 DraI site | AAAAAAACCACCGCTACCAGCGGTGGTTTGTTTGCCGGATCAAGAGCTACCAACTCTTTTTCCGAGGTAACTGGCTTCAGCAGAGCGCAGATACCAAATACTGTTCTTCTAGTGTAGCCGTAGTTAGGCCACCACTTCAAGAACTCTGTAGCACCGCCTACATACCTCGCTCTGCTAATCCTGTTACCAGTGGCTGCTGCCAGTGGCGATAAGTCGTGTCTTACCGGGTTGGACTCAAGACGATAGTTACCGGATAAGGCGCAGCGGTCGGGCTGAACGGGGGGTTCGTGCACACAGCCCAGCTTGGAGCGAACGACCTACACCGAACTGAGATACCTACAGCGTGAGCTATGAGAAAGCGCCACGCTTCCCGAAGGGAGAAAGGCGGACAGGTATCCGGTAAGCGGCAGGGTCGGAACAGGAGAGCGCACGAGGGAGCTTCCAGGGGGAAACGCCTGGTATCTTTATAGTCCTGTCGGGTTTCGCCACCTCTGACTTGAGCATCGATTTTTGTGATGCTCGTCAGGGGGGCGGAGCCTATGGAAAAACGCCAGCAACGCAGAAAGGCCCACCCGAAGGTGAGCCAGGTGATTACATTTAGGTCCTCAAGAAAGGCCCACCCGAAGGTGAGCCAGGTGATTACATTTAGGTCCTCAAGAAAGGCCCACCCGAAGGTGAGCCAGGTGATTACATTTAGGTCCTCAAGAAAGGCCCACCCGAAGGTGAGCCAGGTGATTACATTTAGGTCCTCAAGAAAGGCCCACCCGAAGGTGAGCCAGGTGATTACATTTGGGCCCTCATTACCAATGCTTAATCAGTGAGGCACCTATCTCAGCGATCTGTCTATTTCGTTCATCCATAGTTGCCTGACTCCCCGTCGTGTAGATAACTACGATGCGGGAGGGCTTACCATCTGGCCCCAGTGCTGCAATGATACCGCGAGACCCACGCTCACCGGCTCCAGATTTATCAGCAATAAACCAGCCAGCCGGGAGGGCCGAGCGCAGAAGTGATCCTGCAACTTTATCCGCCTCCATCCAGTCTATTAATTGTTGCCGGGAAGCTAGAGTAAGTAGTTCGCCAGTTAATAGTTTGCGCAACGTTGTTGCCATTGCTACAGGCATCGTGGTGTCACGCTCGTCGTTTGGTATGGCTTCATTCAGCTCCGGTTCCCAACGATCAAGGCGAGTTACATGATCCCCCATGTTGTGCAAAAAAGCGGTTAGCTCCTTCGGTCCTCCGATCGTTGCCAGAAGTAAGTTGGCCGCAGTGTTATCACTCATGGTTATGGCAGCACTGCATAATTCTCTTACTGTCATGCCATCCGTGAGATGCTTTTCTGTGACTGGTGAGTACTCAACCAAGTCATTCTGAGAATAGTGTATGCGGCGACCGAGTTGCTCTTGCCCGGCGTCAATACGGGATAATACCGCGCCACATAGCAGAACTTTAAAAGTGCTCATCATTGGAAAACGTTCTTCGGGGCGTAAACTCTCAAGGATCTTACCGCTGTTGAGATCCAGTTCGATGTAACCCACTCGTGCACCCAACTGATCTTCAGCATCTTTTACTTTCACCAGCGTTTCTGGGTGAGCAAAAACAGGAAGGCAAAATGCCGCAAAAAAGGGAATAAGGGCGACACGGAAATGTTGAATACTCATTTTAGCTTCCTTAGCTCCTGAAAATCTCGATAACTCAAAAAATACGCCCGGTAGTGATCTTATTTCATTATGGTGAAAGTTGGAACCTCTTACGTGCCGATCAAGTCAAAAGCCTCCGGTCGGAGGCTTTTGACTTTCTGCTATGGAGGTCAGGTATGGGTCAGTATTGAGCCTCAGGAAACAGCTATGACATCAAGCTGACTAGATAATCTAGCTGATCGTGGACCGATCATACGTATAATGCCGTAAGATCACGCGCCTTCTCCCTTAGCCTACCGAAGTAGCCCAGGTCGGACCGCGAGGAGGTGGAGATGCCATGCCGACCCTGTCATGGATACGATCGTACTCGGAACCTATAGTGAGTCGTATTAGAGGCCGCATATCATTCAGGACGAGCCTCAGACTCCAGCGTAACTGGACTGCAATCAACTCACTGGCTCACCTTCCGGTCCACGATCAGCTAGAATCAAGCTGACTAGATAAACTGGCCGTCGTTTTACACGGGTGGGCCTTTCTTCGGTAGAAAATCAAAGGATCTTCTTGAGATCCTTTTTTTCTGCGCGTAATCTGCTGCTTGCAAAC | Fig 1C |
| pMT1427, Plasmid with no DraI sites | TTGAGATCCTTTTTTTCTGCGCGTAATCTGCTGCTTGCAAACAAAAAAACCACCGCTACCAGCGGTGGTTTGTTTGCCGGATCAAGAGCTACCAACTCTTTTTCCGAGGTAACTGGCTTCAGCAGAGCGCAGATACCAAATACTGTTCTTCTAGTGTAGCCGTAGTTAGGCCACCACTTCAAGAACTCTGTAGCACCGCCTACATACCTCGCTCTGCTAATCCTGTTACCAGTGGCTGCTGCCAGTGGCGATAAGTCGTGTCTTACCGGGTTGGACTCAAGACGATAGTTACCGGATAAGGCGCAGCGGTCGGGCTGAACGGGGGGTTCGTGCACACAGCCCAGCTTGGAGCGAACGACCTACACCGAACTGAGATACCTACAGCGTGAGCTATGAGAAAGCGCCACGCTTCCCGAAGGGAGAAAGGCGGACAGGTATCCGGTAAGCGGCAGGGTCGGAACAGGAGAGCGCACGAGGGAGCTTCCAGGGGGAAACGCCTGGTATCTTTATAGTCCTGTCGGGTTTCGCCACCTCTGACTTGAGCATCGATTTTTGTGATGCTCGTCAGGGGGGCGGAGCCTATGGAAAAACGCCAGCAACGCAGAAAGGCCCACCCGAAGGTGAGCCAGGTGATTACATTTAGGTCCTCAAGAAAGGCCCACCCGAAGGTGAGCCAGGTGATTACATTTAGGTCCTCAAGAAAGGCCCACCCGAAGGTGAGCCAGGTGATTACATTTAGGTCCTCAAGAAAGGCCCACCCGAAGGTGAGCCAGGTGATTACATTTAGGTCCTCAAGAAAGGCCCACCCGAAGGTGAGCCAGGTGATTACATTTGGGCCCTCATTACCAATGCTTAATCAGTGAGGCACCTATCTCAGCGATCTGTCTATTTCGTTCATCCATAGTTGCCTGACTCCCCGTCGTGTAGATAACTACGATGCGGGAGGGCTTACCATCTGGCCCCAGTGCTGCAATGATACCGCGAGACCCACGCTCACCGGCTCCAGATTTATCAGCAATAAACCAGCCAGCCGGGAGGGCCGAGCGCAGAAGTGATCCTGCAACTTTATCCGCCTCCATCCAGTCTATTAATTGTTGCCGGGAAGCTAGAGTAAGTAGTTCGCCAGTTAATAGTTTGCGCAACGTTGTTGCCATTGCTACAGGCATCGTGGTGTCACGCTCGTCGTTTGGTATGGCTTCATTCAGCTCCGGTTCCCAACGATCAAGGCGAGTTACATGATCCCCCATGTTGTGCAAAAAAGCGGTTAGCTCCTTCGGTCCTCCGATCGTTGCCAGAAGTAAGTTGGCCGCAGTGTTATCACTCATGGTTATGGCAGCACTGCATAATTCTCTTACTGTCATGCCATCCGTGAGATGCTTTTCTGTGACTGGTGAGTACTCAACCAAGTCATTCTGAGAATAGTGTATGCGGCGACCGAGTTGCTCTTGCCCGGCGTCAATACGGGATAATACCGCGCCACATAGCAGAACCTTGAACGTGCTCATCATTGGAAAACGTTCTTCGGGGCGTAAACTCTCAAGGATCTTACCGCTGTTGAGATCCAGTTCGATGTAACCCACTCGTGCACCCAACTGATCTTCAGCATCTTTTACTTTCACCAGCGTTTCTGGGTGAGCAAAAACAGGAAGGCAAAATGCCGCAAAAAAGGGAATAAGGGCGACACGGAAATGTTGAATACTCATTTTAGCTTCCTTAGCTCCTGAAAATCTCGATAACTCAAAAAATACGCCCGGTAGTGATCTTATTTCATTATGGTGAAAGTTGGAACCTCTTACGTGCCGATCAAGTCAAAAGCCTCCGGTCGGAGGCTTTTGACTTTCTGCTATGGAGGTCAGGTATGGGTCAGTATTGAGCCTCAGGAAACAGCTATGACATCAAGCTGACTAGATAATCTAGCTGATCGTGGACCGATCATACGTATAATGCCGTAAGATCACGCGCCTTCTCCCTTAGCCTACCGAAGTAGCCCAGGTCGGACCGCGAGGAGGTGGAGATGCCATGCCGACCCTGTCATGGATACGATCGTACTCGGAACCTATAGTGAGTCGTATTAGAGGCCGCATATCATTCAGGACGAGCCTCAGACTCCAGCGTAACTGGACTGCAATCAACTCACTGGCTCACCTTCCGGTCCACGATCAGCTAGAATCAAGCTGACTAGATAAACTGGCCGTCGTTTTACACGGGTGGGCCTTTCTTCGGTAGAAAATCAAAGGATCTTC | Fig 1C |
| Fluoro- RapidAlt  (dsDNA Canonical cut site top strand) | 5′‑**Cy5**‑TCTAATACGACTCACTATAGGCACCATCGTTTAAAAAGTCGCACATATCGAGGTGGACATGCATCACGTCTCGTACTTCGAGATGAGCGT-**6-FAM** | Fig 1D-E; Fig 2; Fig 3A-C; Fig 6A-C; Fig 7A; Supp Figs 1,2,3,4,5, and 9. |
| Fluoro- dsDNA Canonical cut site bottom strand | 5′‑**Cy5**‑ACGCTCATCTCGAAGTACGAGACGTGATGCATGTCCACCTCGATATGTGCGACTTTTTAAACGATGGTGCCTATAGTGAGTCGTATTAGA-**6-FAM** | Fig 1D-F; Fig 2A; Fig 6A-C; Fig 7A; Supp Figs 1,2, and 9. |
| RNA - RapidAlt | 5′‑TCTAATACGACTCACTATAGGCACCATCGTTTAAAAAGTCGCACATATCGAGGTGGACATGCATCACGTCTCGTACTTCGAGATGAGCGT-**6-FAM** | Fig 1F |
| ii. Scrambled RapidAlt cut site | 5′‑**Cy5**‑TCTAATACGACTCACTATAGGCACCATCGTTTAAAAAGTCGCACAGCGCCTAGTGGACATGCATCACGTCTCGTACTTCGAGATGAGCGT-**6-FAM** | Fig 3A |
| iii. Scrambled Canonical cut site | 5′‑**Cy5**‑TCTAATACGACTCACTATAGGCACCATCGCACAGGTTGTCGCACATATCGAGGTGGACATGCATCACGTCTCGTACTTCGAGATGAGCGT-**6-FAM** | Fig 3A |
| iv. 5’ Shift 15 nt | 5′‑**Cy5**‑TATAGGCACCATCGTTTAAAAAGTCGCACATATCGAGGTGGACATGCATCACGTCTCGTACTTCGAGATGAGCGTTCTAATACGACTCAC-**6-FAM** | Fig 3A |
| v. 3’ 15 nt del | 5′‑**Cy5**‑TCTAATACGACTCACTATAGGCACCATCGTTTAAAAAGTCGCACATATCGAGGTGGACATGCATCACGTCTCGTA-**6-FAM** | Fig 3A |
| vi. PolyA | 5′‑**Cy5**‑AAAAAAAAAAAAAAAAAAAATATCGAGGAAAAAAAAAAAAAAA-**6-FAM** | Fig 3A |
| MinHP | AAAAAGTCGCACATATCGAAGTGGCTCGTACTTCGAGATGTGC | Fig 8A-C;  Supp Figs 6, 11, 12, and 14. |
| Fluoro-MinHP | 5′‑**Cy5**‑AAAAAGTCGCACATATCGAAGTGGCTCGTACTTCGAGATGTGC-**6-FAM** | Fig 3B-D;  Fig 7B-E;  Supp Figs 4,5, and 10. |
| Min HP + Wobble | 5’-AAAAAGTCGCACATATCGAGGTGGCTCGTACTTCGAGATGTGC-3’ | Fig 4A,B; Fig 5A; Supp Fig 6 |
| Fluoro-Min HP + Wobble | 5′‑**Cy5**‑AAAAAGTCGCACATATCGAGGTGGCTCGTACTTCGAGATGTGC-**6-FAM** | Fig 3B |
| GG Mismatch | AAAAAGTCGCACATGTCGAGGTGGCTCGTACTTCGAGATGTGC | Fig 4A |
| GA Mismatch | AAAAAGTCGCACATGTCGAGGTGGCTCGTACTTCGAAATGTGC | Fig 4A; Supp Fig 6 |
| AA Mismatch | AAAAAGTCGCACATATCGAGGTGGCTCGTACTTCGAAATGTGC | Fig 4A |
| CA Mismatch | AAAAAGTCGCACATCTCGAGGTGGCTCGTACTTCGAAATGTGC | Fig 4A |
| AC Mismatch | AAAAAGTCGCACATATCGAGGTGGCTCGTACTTCGACATGTGC | Fig 4A |
| CC Mismatch | AAAAAGTCGCACATCTCGAGGTGGCTCGTACTTCGACATGTGC | Fig 4A |
| TT Mismatch | AAAAAGTCGCACATTTCGAGGTGGCTCGTACTTCGATATGTGC | Fig 4A |
| TC Mismatch | AAAAAGTCGCACATTTCGAGGTGGCTCGTACTTCGACATGTGC | Fig 4A |
| CT Mismatch | AAAAAGTCGCACATCTCGAGGTGGCTCGTACTTCGATATGTGC | Fig 4A |
| TG Mismatch | AAAAAGTCGCACATTTCGAGGTGGCTCGTACTTCGAGATGTGC | Fig 4A; Supp Fig 6 |
| GT Mismatch | AAAAAGTCGCACATGTCGAGGTGGCTCGTACTTCGATATGTGC | Fig 4A; Supp Fig 6 |
| UG Mismatch | AAAAAGTCGCACATUTCGAGGTGGCTCGTACTTCGAGATGTGC | Fig 4A; Supp Fig 6 |
| GU Mismatch | AAAAAGTCGCACATGTCGAGGTGGCTCGTACTTCGAUATGTGC | Fig 4A; Supp Fig 6 |
| CG Mismatch | AAAAAGTCGCACATCTCGAGGTGGCTCGTACTTCGAGATGTGC | Fig 4A |
| GC Mismatch | AAAAAGTCGCACATGTCGAGGTGGCTCGTACTTCGACATGTGC | Fig 4A |
| MinHP GA Mismatch | AAAAAGTCGCACATGTCGAAGTGGCTCGTACTTCGAAATGTGC | Fig 4A; Supp Fig 6 |
| MinHP TG Wobble | AAAAAGTCGCACATTTCGAAGTGGCTCGTACTTCGAGATGTGC | Fig 4A; Supp Fig 6 |
| MinHP GT  Wobble | AAAAAGTCGCACATGTCGAAGTGGCTCGTACTTCGATATGTGC | Fig 4A; Supp Fig 6 |
| MinHP UG Wobble | AAAAAGTCGCACATUTCGAAGTGGCTCGTACTTCGAGATGTGC | Fig 4A; Supp Fig 6 |
| MinHP GU Wobble | AAAAAGTCGCACATGTCGAAGTGGCTCGTACTTCGAUATGTGC | Fig 4A; Supp Fig 6 |
| CG Cut Site  (MinHP with one bp change) | AAAAAGTCGCACATATCGAGGTGGCTCGTACCTCGAGATGTGC | Fig 4B; Fig 5B |
| CC Cut Site | AAAAAGTCGCACATATCCAGGTGGCTCGTACCTGGAGATGTGC | Fig 4B |
| GG Cut Site | AAAAAGTCGCACATATGGAGGTGGCTCGTACCTCCAGATGTGC | Fig 4B |
| GC Cut Site | AAAAAGTCGCACATATGCAGGTGGCTCGTACCTGCAGATGTGC | Fig 4B |
| CA Cut Site | AAAAAGTCGCACATATCAAGGTGGCTCGTACCTTGAGATGTGC | Fig 4B |
| CT Cut Site | AAAAAGTCGCACATATCTAGGTGGCTCGTACCTAGAGATGTGC | Fig 4B |
| GA Cut Site | AAAAAGTCGCACATATGAAGGTGGCTCGTACCTTCAGATGTGC | Fig 4B |
| GT Cut Site | AAAAAGTCGCACATATGTAGGTGGCTCGTACCTACAGATGTGC | Fig 4B |
| AC Cut Site | AAAAAGTCGCACATATACAGGTGGCTCGTACCTGTAGATGTGC | Fig 4B |
| AG Cut Site | AAAAAGTCGCACATATAGAGGTGGCTCGTACCTCTAGATGTGC | Fig 4B |
| TC Cut Site | AAAAAGTCGCACATATTCAGGTGGCTCGTACCTGAAGATGTGC | Fig 4B |
| TG Cut Site | AAAAAGTCGCACATATTGAGGTGGCTCGTACCTCAAGATGTGC | Fig 4B |
| AA Cut Site | AAAAAGTCGCACATATAAAGGTGGCTCGTACCTTTAGATGTGC | Fig 4B |
| AT Cut Site | AAAAAGTCGCACATATATAGGTGGCTCGTACCTATAGATGTGC | Fig 4B |
| TA Cut Site | AAAAAGTCGCACATATTAAGGTGGCTCGTACCTTAAGATGTGC | Fig 4B |
| TT Cut Site | AAAAAGTCGCACATATTTAGGTGGCTCGTACCTAAAGATGTGC | Fig 4B |
| CU-GG Cut Site | AAAAAGTCGCACATATCUAGGTGGCTCGTACCTGGAGATGTGC | Fig 4B |
| UG-CG Cut Site | AAAAAGTCGCACATATUGAGGTGGCTCGTACCTCGAGATGTGC | Fig 4B |
| CG-UG Cut Site | AAAAAGTCGCACATATCGAGGTGGCTCGTACCTUGAGATGTGC | Fig 4B |
| UG-UG Cut Site | AAAAAGTCGCACATATUGAGGTGGCTCGTACCTUGAGATGTGC | Fig 4B |
| GG-CU Cut Site | AAAAAGTCGCACATATGGAGGTGGCTCGTACCTCUAGATGTGC | Fig 4B |
| mCG-TT Cut Site | AAAAAGTCGCACATATmCGAGGTGGCTCGTACTTTTAGATGTGC | Fig 4B |
| CG-mCG Cut Site | AAAAAGTCGCACATATCGAGGTGGCTCGTACTTmCGAGATGTGC | Fig 4B |
| mCG-mCG Cut Site | AAAAAGTCGCACATATmCGAGGTGGCTCGTACTTmCGAGATGTGC | Fig 4B |
| TG-CG Cut Site | AAAAAGTCGCACATATTGAGGTGGCTCGTACCTCGAGATGTGC | Fig 4B |
| CG-TG Cut Site | AAAAAGTCGCACATATCGAGGTGGCTCGTACCTTGAGATGTGC | Fig 4B |
| TG-TG Cut Site | AAAAAGTCGCACATATTGAGGTGGCTCGTACCTTGAGATGTGC | Fig 4B |
| MS 2-mismatch hairpin | TGCGCACATATCGAGGTGGCTCGTACTTCGAGATGAGCGCT | Fig 5C |
| Canonical Hairpin | 5′‑**Cy5**‑AAAAAGTCGCACTTTTTAAAGTGGCTCGTACTTTAAAAAGTGC-**6-FAM** | Fig 7B-E;  Supp Fig 10. |
| Perfect Duplex Bottom | ACGCTCATCTCGAAGTACGAGACGTGATGCATGTCCACCTCGATATGTGCGACTTTTTAAACGATGGTGCCTATAGTGAGTCGTATTAGA | Fig 7A |
| Mismatch Duplex Bottom | ACGCTCATCTCGAAGTACGAGACGTGATGCATGTCCACCTCGAGATGTGCGACTTTTTAAACGATGGTGCCTATAGTGAGTCGTATTAGA | Fig 7A |
| vii – T-Rich Overhang | GCTTTTTTTGCACATATCGAAGTGGCTCGTACTTCGAGATGTGC | Fig 8A,  Supp Fig 11 |
| viii – Alt  T-Rich Overhang | GCAGTTTTTGCACATATCGAAGTGGCTCGTACTTCGAGATGTGC | Fig 8A,  Supp Fig 11 |
| ix – 8T Overhang | TTTTTTTTGCACATATCGAAGTGGCTCGTACTTCGAGATGTGC | Fig 8A,  Supp Fig 11 |
| x – 8A Overhang | AAAAAAAAGCACATATCGAAGTGGCTCGTACTTCGAGATGTGC | Fig 8A,  Supp Fig 11 |
| xi – 8C Overhang | CCCCCCCCGCACATATCGAAGTGGCTCGTACTTCGAGATGTGC | Fig 8A,  Supp Fig 11 |
| xii – 8G Overhang | GGGGGGGGGCACATATCGAAGTGGCTCGTACTTCGAGATGTGC | Fig 8A,  Supp Fig 11 |
| xiii – 3’ Overhang | GCACATATCGAAGTGGCTCGTACTTCGAGATGTGCTGTAAAAA | Fig 8B,  Supp Fig 12 |
| xiv – No Overhang | GCACATATCGAAGTGGCTCGTACTTCGAGATGTGC | Fig 8B,  Supp Fig 12 |
| xv – Long Overhang | AAAAAAAAAAGTCGCACATATCGAAGTGGCTCGTACTTCGAGATGTGC | Fig 8B,  Supp Fig 12 |
| xvi – +5 bp Down | AAAAAAAAAAGTCGCACATATCGAAGTGGCTCGTACTTCGAGATGTGC | Fig 8C,  Supp Fig 14 |
| xvii – +5 bp Up-stream | AAAAAAAAAAGTCGCACATATCGAAGTGGCTCGTACTTCGAGATGTGC | Fig 8C,  Supp Fig 14 |
| Abasic (“idSp” is IDT code for abasic) | **6-FAM**-CCGCACATATCGAGGTGGCTCGTACCTCGA/idSp/ATGTGCGG | Supp Fig 3 |
| 1./7. Top | **6-FAM**-TAACCCTATCGACGTAGATCGAGTT | Supp Fig 7A-C |
| 2./8. Top | **6-FAM**-GTGGATATCTCTAGACCCTCCATGT | Supp Fig 7A-C |
| 3./9. Top | **6-FAM**-AACAACTGTTCTCCGCAAATGCCAC | Supp Fig 7A-C |
| 4./10. Top | **6-FAM**-GAATTAACTATAAGGGTCCGTTCGG | Supp Fig 7A-C |
| 5./10. Top | **6-FAM**-ACTCTCGATCGACAGGCGGTACTTG | Supp Fig 7A-C |
| 6./11. Top | **6-FAM**-AAGTCGCACATATTGAGGTGGCTCG | Supp Fig 7A-C |
| 1. Bottom | AACTCGATCTACGGCGATAG | Supp Fig 7A,C |
| 2. Bottom | ACATGGAGGGTCTGGAGATA | Supp Fig 7A,C |
| 3. Bottom | GTGGCATTTGCGGGGAACAG | Supp Fig 7A,C |
| 4. Bottom | CCGAACGGACCCTGATAGTT | Supp Fig 7A,C |
| 5. Bottom | CAAGTACCGCCTGGCGATCG | Supp Fig 7A,C |
| 6. Bottom | CGAGCCACCTCGAGATGTGC | Supp Fig 7A,C |
| 7. Bottom | AACTCGATCTACGTCGATAG | Supp Fig 7A,B |
| 8. Bottom | ACATGGAGGGTCTAGAGATA | Supp Fig 7A,B |
| 9. Bottom | GTGGCATTTGCGGAGAACAG | Supp Fig 7A,B |
| 10. Bottom | CCGAACGGACCCTTATAGTT | Supp Fig 7A,B |
| 11. Bottom | CAAGTACCGCCTGTCGATCG | Supp Fig 7A,B |
| 12. | **Cy5**-AAGTCGCACATATCGAGGTGGCTCG | Supp Fig 13 |
| 13. Top | CGAGCTACTTCGAGATGTGC | Supp Fig 13 |
| 14. Top | CGAGCTACTTCGAGATGTGCGACTT | Supp Fig 13 |
| 15. Top | CGAGCCACCTCGAGATGTGC | Supp Fig 13 |
| 16. Top | CGAGCCACCTCGAGATGTGCGACTT | Supp Fig 13 |
| 17. Top | CGAGCCACCTCGATATGTGCGACTT | Supp Fig 13 |

## Table S2: Sequencing results

**L1 EN WT on Duplex DNA**

| **Bottom Strand (5’ to 3’) - Sequence Upstream of Cut Site (1% cutoff)** | **Percent** |
| --- | --- |
| ACGCTCATCTCGAAGTACGAGACGTGATGCATGTCCACCTCGATATGTGCGACTTTTTA | 13.6 |
| ACGCTC | 10.5 |
| ACGCTCATCTCGAAGTACGAGACGTGATGCATGTCCACCTCGATATGTGCGACTTTTT | 8.0 |
| ACGCTCATCTCGAAGTACGAGACGT | 6.2 |
| ACGCTCATCTCGAAGTACGAGACGTGATGCATGTCCACCTC | 6.0 |
| ACGCTCATCTCGAAGTACGAGACGTGATGCATGTCCACCTCGATATGTGCGACTTTTTAAACG | 4.9 |
| ACGCTCATCTC | 4.5 |
| ACGCTCATCTCGA | 3.8 |
| ACGCTCATCTCGAAGTACGAGACGTGATGC | 3.0 |
| ACGCTCATCTCGAAGTACGAGACGTGATGCATGTCCACCTCGA | 2.8 |
| ACGCTCATCTCGAAGTACGAGACGTGATGCATGTCC | 2.5 |
| ACGCTCATCTCGAAGTACGAGACGTGATGCATGTCCACCTCGATATGTGCGACTTTTTAA | 2.5 |
| ACGCTCATCTCGAAGTACGAGACGTGATGCATGTCCACCTCGATATGTGCGACTTTTTAAAC | 2.1 |
| ACGCTCATCTCGAAGTACGAGACGTGATGCATGTCCACCTCGAT | 1.9 |
| ACGCTCATCTCGAAGTACGAGACGTGATGCATGTCCACCTCGATATGTGCGACTTTT | 1.8 |
| ACGCTCATCTCGAAGTACGA | 1.8 |
| ACGCTCATCTCGAAGTACGAGACGTGATGCATGTCCA | 1.7 |
| ACGCTCATCTCGAAGTACGAGACGTGATGCATGTCCACCTCGATATGTGCGACTTTTTAAACGATGGT | 1.6 |
| ACGCTCATCTCGAAGTACGAGACGTGATGCATGTCCACCTCG | 1.6 |
| ACGCTCATCTCGAAGTACGAGACGTGATGCATGTC | 1.2 |
| ACGCTCATCTCG | 1.2 |
| ACG | 1.1 |

| **Top Strand (5’ to 3’) - Sequence Upstream of Cut Site (1% cutoff)** | **Percent** |
| --- | --- |
| TCTAATACGACTCACTATAGGCACCATCGTTTAAAAAGTCGCACATATCGAGGTGGACATGCATCACGTCTC | 31.0 |
| TCTAATACGACTCACTATAGGCACCATCGTTTAAAAAGTCGCACATATCGAGGTGGACATGCATCAC | 9.4 |
| TCTAATACGACTCACTATAGGCACCATCGTTTAAAAAGTCGC | 8.8 |
| TCTAATACGACTCACTATAGGCACCATCGTTTAAAAAGTCGCACATATCGAGGTGGACATGC | 6.6 |
| TCTAATACGACTCACTATAGGCACCATCGTTTAAAAAGTCGCACATATCGAGGTG | 5.2 |
| TCTAATACGACTCACTATAGGCACCATCGTTTAAAAAGTCGCACATATCGA | 4.7 |
| TCTAATACGACTCACTATAGGCACCATCGTTTAAAAAGTCGCACATATCGAGGTGGACATGCATCACG | 4.0 |
| TCTAATACGACTCACTATAGGCACCATCGTTTAAAAAGTCGCACATATCGAGGTGG | 3.5 |
| TCTAATACGACTCACTATAGGCACCATCGTTTAAAAAGTCGCACATATCGAGGTGGACATGCATCACGT | 1.9 |
| TCTAATACGACTCACTATAGGCACCATCGTTTAAAAAGTCGCACATATC | 1.7 |
| TCTAATACGACTCACTATAGGCACCATCGTTTAAAAAGTCGCACATATCGAGGTGGACATGCATC | 1.7 |
| TCTAATACGACTCACTATAGGCACCATCGTTTAAAAAGTCGCACATATCGAGGTGGAC | 1.5 |
| TCTAATACGACTCACTATAGGCACCATCGTTTAAAAAGTC | 1.4 |
| TCTAATACGACTCACTATAGGCACCATCGT | 1.2 |
| TCTAATACGACTCACTATAGGCACCATCGTTTAAAAAGTCGCACATATCGAGGTGGA | 1.1 |

**L1 EN D145N, E43S mutant on Duplex DNA**

| **Bottom Strand (5’ to 3’) - Sequence Upstream of Cut Site (2% cutoff)** | **Percent** |
| --- | --- |
| ACGCTCATCTCGAAGTACGAGACGTGATGCATGTCCACCTCGATATGTGCGACTTTTTAAACG | 7.9 |
| ACGCTCATCTCGAAGTACGAGACGTGATGCATGTCCACCTCGATATGTGCGA | 6.9 |
| ACG | 6.8 |
| ACGCTCATCTCGAAGTACGAGACGTGATGCATGTCCACCTCGATATGTGCGACTTTTTAA | 5.0 |
| ACGCTCATCTCGAAGTACGAGACGTGATGCATGTCCACCTCGATATGTGCGACTTTTTAAA | 4.4 |
| ACGCTCATCTCGAAGTACGAGACGTGATGCATGTCCACCTCG | 4.0 |
| ACGCTCATCTCGAAGTACGAGACGTGATGCATGTCCACCTCGATATGTGCG | 3.8 |
| ACGCTCATCTCGAAGTACGAGACGTGATGCATGTCCACCTCGATATGTG | 3.6 |
| ACGCTCATCTCGAAGTACGAGACGTGATGCATGTCCACCTCGATATGTGCGACTTTTTA | 3.2 |
| ACGCTCATCTCGAAGTACGAGACGTGATGCATGTCCACCTCGATATGTGCGACTTTTTAAACGA | 2.7 |
| ACGCTCATCTCGAAGTACGAGACGTGATG | 2.7 |
| ACGCTCATCTCGAAGTACGAGACGTGATGCATG | 2.4 |
| ACGCTCATCTCGAAGTACGAGACGTGATGCATGTCCA | 2.3 |
| ACGCTCATCTCGAAGTACGAGACGTGATGCATGTCCACCTCGATATGTGCGACTTTTTAAACGAT | 2.3 |
| ACGCTCATCTCGAAGTACGAGACGTGATGCATGTCCACCTCGATA | 2.2 |
| ACGCTCATCTCGAAGTACGAGACGTGATGCATGTCCACCTCGATATGTGCGACTTTTTAAAC | 2.1 |

| **Top Strand (5’ to 3’) - Sequence Upstream of Cut Site (2% cutoff)** | **Percent** |
| --- | --- |
| TCTAATACGACTCACTATAGGCACCATCGTTTAAAAAGTCGCACATATCGAGGTGGACATGCATCACG | 18.5 |
| TCTAATACGACTCACTATAGGCACCATCGTTTAAAAAGTCGCACATATCGAGGTGGACATG | 7.0 |
| TCTAATACGACTCACTATAGGCACCATCGTTTAAAAAGTCGCACATATCGAGGTGG | 6.1 |
| TCTAATACGACTCACTATAGGCACCATCGTTTAAAAAGTCGCACATATCGAGGTG | 4.6 |
| TCTAATACGACTCACTATAGG | 4.0 |
| TCTAATACG | 4.0 |
| TCTAATACGACTCACTATAGGCACCATCGTTTAAAAAGTCGCACATATCGAGGTGGACATGCA | 2.8 |
| TCTAATACGACTCACTATAGGCACCATCGTTTAAAAAGTCGCACATATCGAGGTGGACATGCATCA | 2.6 |
| TCTAATACGACTCACTATAGGCACCATCGTTTAAAAAG | 2.6 |
| TCTAATACGACTCACTATAGGCACCATCGTTTAAAAAGTCGCACATATCGAGGTGGA | 2.6 |
| TCTAATACGACTCACTATAGGCACCATCG | 2.4 |
| TCTAATACGACTCACTATAGGCACCATCGTTTAAAAAGTCG | 2.4 |
| TCTAATACGACTCACTATAGGCACCATCGTTTAAAAAGTCGCACATATCGAGGTGGACATGCATCACGT | 2.2 |
| TCTAATACGA | 2.1 |

**L1 EN WT on RapidAlt Substrate**

| **Sequence Upstream of Cut Site (5’ to 3’) (1% cutoff)** | **Percent** |
| --- | --- |
| TCTAATACGACTCACTATAGGCACCATCGTTTAAAAAGTCGCACATATC | 68.2 |
| TCTAATACGACTCACTATAGGCACCATCGTTTAAAAAGTCGCACATATCGAGGTGGACATGCATCACGTCTC | 10.7 |
| TCTAATACGACTCACTATAGGCACCATCGTTTAAAAAGTCGCAC | 6.2 |
| TCTAATACGACTCACTATAGGCACCATCGTTTAAAAAGTCGCACATATCGAGGTGGACATGCATCACG | 2.1 |
| TCTAATACGACTCACTATAGGCACCATCGTTTAAAAAGTCGCACATATCGAGGTGGACATGCATCACGTC | 1.5 |
| TCTAATACGACTCACTATAGGCACCATCGTTTAAAAAGTCGCACATATCGAGGTGGACATGCATCACGT | 1.3 |

**L1 EN D145N, E43S mutant on RapidAlt Substrate**

| **Sequence Upstream of Cut Site (5’ to 3’) (2% cutoff)** | **Percent** |
| --- | --- |
| TCTAATACGACTCACTATAGGCACCATCGTTTAAAAAGTCGCACATATCGAGGTGGACATGCATCACG | 18.0 |
| TCTAATACGACTCACTATAGGCACCATCGTTTAAAAAGTCGCACATATCGAGGTG | 7.3 |
| TCTAATACGACTCACTATAGGCACCATCGTTTAAAAAGTCGCACATATCGAGGTGG | 7.0 |
| TCTAATACGACTCACTATAGGCACCATCGTTTAAAAAGTCGCACATATCGAGGTGGACATG | 7.0 |
| TCTAATACGACTCACTATAGG | 4.4 |
| TCTAATACG | 3.8 |
| TCTAATACGACTCACTATAGGCACCATCGTTTAAAAAGTCGCACATATCGAGG | 2.9 |
| TCTAATACGACTCACTATAGGCACCATCGTTTAAAAAG | 2.7 |
| TCTAATACGACTCACTATAGGCACCATCGTTTAAAAAGTCG | 2.3 |
| TCTAATACGACTCACTATAGGCACCATCGTTTAAAAAGTCGCACATATCGAGGTGGACATGCATCA | 2.3 |
| TCTAATACGACTCACTATAGGCACCATCG | 2.3 |
| TCTAATACGACTCACTATAGGCACCATCGTTTAAAAAGTCGCACATATCGAGGTGGACATGCATCACGT | 2.1 |

## Table S3: Dinucleotide frequencies at L1 insertion breakpoints

| **Positive sense insertions** | | | **Negative sense insertions** | | |
| --- | --- | --- | --- | --- | --- |
| **1st strand cut dinucleotide frequencies:** | **Number** | **Percent** | **1st strand cut dinucleotide frequencies:** | **Number** | **Percent** |
| TA | 4426 | 62.5 | TA | 4532 | 64.3 |
| CA | 935 | 13.2 | TG | 975 | 13.8 |
| GA | 615 | 8.7 | CA | 559 | 7.9 |
| TG | 469 | 6.6 | AA | 366 | 5.2 |
| TT | 281 | 4.0 | TC | 343 | 4.9 |
| AA | 149 | 2.1 | GA | 57 | 0.8 |
| CT | 56 | 0.8 | AG | 49 | 0.7 |
| TC | 36 | 0.5 | TT | 32 | 0.5 |
| AC | 26 | 0.4 | AT | 27 | 0.4 |
| AT | 26 | 0.4 | GG | 24 | 0.3 |
| AG | 20 | 0.3 | AC | 23 | 0.3 |
| GG | 13 | 0.2 | CC | 22 | 0.3 |
| CC | 11 | 0.2 | CT | 11 | 0.2 |
| GT | 11 | 0.2 | CG | 9 | 0.1 |
| CG | 6 | 0.08 | GC | 9 | 0.1 |
| GC | 2 | 0.03 | GT | 6 | 0.09 |
| **YpR cut sites: 5836 / 7082** | **Percent YpR: 82** | | **YpR cut sites: 6075 / 7044** | **Percent YpR: 86** | |
| **Positive sense insertions** | | | **Negative sense insertions** | |  |
| **2nd strand cut dinucleotide frequencies:** | **Number** | **Percent** | **2nd strand cut dinucleotide frequencies:** | **Number** | **Percent** |
| TT | 1599 | 22.6 | AA | 1538 | 21.8 |
| TA | 782 | 11.0 | TA | 834 | 11.8 |
| TG | 612 | 8.6 | AG | 597 | 8.5 |
| CT | 603 | 8.5 | CA | 584 | 8.3 |
| AA | 546 | 7.7 | TT | 550 | 7.8 |
| AT | 527 | 7.4 | AT | 508 | 7.2 |
| TC | 483 | 6.8 | GA | 461 | 6.5 |
| CA | 414 | 5.8 | TG | 429 | 6.1 |
| AG | 319 | 4.5 | CT | 320 | 4.5 |
| CC | 243 | 3.4 | GT | 263 | 3.7 |
| GT | 232 | 3.3 | GG | 226 | 3.2 |
| AC | 221 | 3.1 | TC | 221 | 3.1 |
| GA | 214 | 3.0 | AC | 199 | 2.8 |
| GC | 125 | 1.8 | GC | 145 | 2.1 |
| GG | 121 | 1.7 | CC | 127 | 1.8 |
| CG | 41 | 0.6 | CG | 42 | 0.6 |
| **YpR cut sites: 1849 / 7082** | **Percent YpR: 27** | | **YpR cut sites: 1889 / 7044** | **Percent YpR: 27** | |
